# TL-CRANN: a transfer-learning-based hybrid CNN-RNN model with attention mechanism for cross-domain IoT attack detection

**DOI:** 10.3389/frai.2026.1934604

**Published:** 2026-09-29

**Authors:** L. Sankar Ganesh, P. Mohan Kumar

**Affiliations:** School of Computer Science and Engineering (SCOPE), Vellore Institute of Technology, Vellore, Tamil Nadu, India

**Keywords:** attention mechanism, CNN-RNN hybrid model, cross-domain attack detection, deep learning, IoT security, transfer-learning

## Abstract

**Introduction:**

The rapid and pervasive growth of Internet of Things (IoT) devices has significantly expanded the attack surface of current network environments, making them vulnerable to advanced and evolving cyber threats. However, current intrusion detection systems (IDS) usually suffer from weak cross-domain generalization, high false positive rates, and poor performance against novel attacks.

**Methods:**

To overcome these issues, this paper proposes TL-CRANN, a transfer-learning-based hybrid deep learning framework utilizing a convolutional neural network (CNN), bidirectional long short-term memory (Bi-LSTM), and a multi-head self-attention mechanism for cross-domain IoT attack detection. Transfer learning is leveraged to transfer knowledge acquired from source-domain traffic and achieve rapid adaptation to heterogeneous target IoT environments with scarce labeled data. The proposed model is evaluated using three benchmark datasets: CICIDS2017, TON_IoT, and Bot-IoT.

**Results:**

Experimental results show that TL-CRANN achieved detection accuracies of 98.37%, 96.21%, and 97.14%, respectively, on the three datasets, and significantly outperformed existing state-of-the-art machine learning and deep learning baselines.

**Discussion:**

The results show that transfer learning improves the cross-domain detection performance and training cost. Moreover, the ablation analysis shows that CNN, Bi-LSTM and attention mechanisms-based modules are contribution to effectiveness of proposed framework which also confirms the applicability of TL-CRANN for cross-domain IoT attack detection.

## 1 Introduction

### 1.1 Background and motivation

Internet of Things (IoT) devices are now increasingly embedded in smart homes, healthcare systems, industrial automation, smart cities, and critical infrastructure, having fundamentally altered connectivity. It is estimated that over 29.4 billion connected IoT devices are forecast for 2030 (Yalli et al., 2024) and this has given rise to huge volumes of varied network traffic that traverse heterogeneous networks, using different protocols. IoT technology enables the improvement of efficiency, scalability, and automation while being inherently resource-constrained in its IoT endpoints which implies constrained processing, memory size and no built-in security mechanisms. These characteristics make the IoT endpoints vulnerable to advanced cyber-threats (Sahu, 2024; Altaibek et al., 2025).

Recent cyber-attacks against IoT environments are increasing rapidly in scale, in sophistication, and in inter-domain impact. Critical incidents like the Mirai botnet attack in 2016, where thousands of compromised IoT devices were utilized to perpetrate a 1.1 Tbps DDoS attack against Dyn's DNS infrastructure and more recent campaigns such as Bricker Bot and Hajime further confirm the danger of IoT-based attacks (Gelgi et al., 2024). Exploitation of protocol specific security flaws within the IoT communication stack such as MQTT, CoAP and Zigbee networks has also allowed such attacks to become domain-spanning, targeting numerous devices within the wider network (Husnain et al., 2022; Ferrag et al., 2022).

Recent developments in Artificial Intelligence of Things (AIoT), Industrial IoT (IIoT), and edge computing have further complicated the IoT network environment by producing extremely diverse, and ever-changing traffic patterns. Therefore, the expectations of the modern intrusion detection system have to be not only a high detection accuracy but also cross-domain generalization, high scalability, low-latency inference, and robustness toward unknown and emerging cyber threats. As reported in recent surveys by Al-Haija and Droos (2025) and Mallidi and Ramisetty (2025), these features are prominent research challenges for next generation IoT intrusion detection systems, especially in the context of large-scale heterogeneous environments.

Traditional IDS-signature based and anomaly based-have struggled to effectively manage the highly dynamic, heterogeneous, and high-throughput characteristics of IoT network traffic. Signature based IDS cannot identify zero-day attacks, and rule-based anomaly detection systems suffer from high false positive rates which makes them unusable in a real-world deployment scenario (Isong et al., 2024). The various methods for machine learning-based IDS show great potential but most suffer from a severe decline in detection when tested in an environment different than their training environment-which makes them unsuitable for actual IoT applications (Elangovan et al., 2026). Machine Learning (ML) and Deep Learning (DL) approaches have greatly improved the state of IoT Intrusion detection, but existing DL based IDS models lack the multi domain generalizability, they are mostly built for a specific IoT environment (Somasundaram, 2020).

Transfer-learning (TL) has been an unprecedented approach to deep learning that provides a way to fine-tune a model pre-trained on a large dataset to a target task that has limited labeled data. For IoT security applications, TL provides a plausible way to cross-domain and share knowledge, as the model pre-trained on a domain-rich labeled dataset can be tuned to unseen attack scenarios with much lower retraining cost (Zhuang et al., 2020b). Nevertheless, the utilization of TL to Hybrid Spatiotemporal architectures are relatively absent from the IoT intrusion detection literature (Mahdavi et al., 2022).

While transfer-learning has greatly facilitated knowledge reuse among heterogeneous IoT datasets, the recent result shows that transferring merely convolutional or recurrent representations rends to underfit when facing large domain shifts. Transfer-learning with attention mechanisms can facilitate feature discrimination as Abdelhamid et al. (2024), and a recent summary of reviews highlight that lightweight hybrid models based on transfer-learning, modeling of temporal sequences and attention mechanisms can provide more robust cross-domain applicability with less computational complexity for IoT devices limited by resources.

In response to the foregoing motivations, in this work, we present a novel deep learning framework, TL-CRANN (Transfer-learning-based CNN-RNN with Attention Neural Network), in an attempt to develop a reliable, scalable and cross-domain approach for IoT attack detection by effectively integrate the strengths of CNNs to perform spatial feature extraction on network flow features, LSTMs to achieve the temporal modeling on sequential network traffic flows and a multi-head self-attention mechanism to enhance the distinguishing capability for the security-sensitive temporal information.

Current deep learning based IoT IDS have three critical drawbacks: (i) Domain dependence- models trained on one IoT environment such as smart home does not perform well on other IoT environments like industrial IoT. (ii) Time blindness- feed forward or convolutional based models can't effectively characterize temporal dynamics of attack sequence such as slow rate DDoS, multi-stages APT attack campaign. (iii) Inefficiency of features-high computational overheads and substandard detection efficiency models that utilize high-dimensional flow features or raw packets with non-hierarchical feature selection process. The proposed TL-CRANN alleviates all three drawbacks.

### 1.2 Research gap and novel contributions

Despite the substantial advance of deep learning for IoT intrusion detection, some challenges prevent current models from generalizing to various IoT scenarios. A number of studies, however, combine CNNs, recurrent networks, attention mechanisms, and transfer-learning to enhance detection performance, but clear methodological reasoning for the combination of the mechanisms is not given. Therefore, it is still not clear in what way each building block mitigates shortcomings of existing model and why their composition results in additional, complementary capabilities rather than an (*ad hoc*) amalgamation of established tools. To overcome this, the novel TL-CRANN approach is proposed based on addressing three major constraints of existing IoT intrusion detection systems.

#### 1.2.1 Limited cross-domain adaptation

The current TL-based IDSs enhance adaptation with a small number of labeled data in the target-domain but they usually transfer the convolution or the recurrent representation alone, which gives restricted guidance for IoT traffic from its heterogeneous nature. TL-CRANN overcomes this limitation via a selective layer freezing technique, while adapting higher-level CNN layers, the Bi-LSTM, and the attention module to capture the target-domain specific attack features, all the while keeping the transferable low-level CNN features as is.

#### 1.2.2 Incomplete integration of spatial, temporal, and attention learning

With CNN-based models, spatial features can be successfully extracted, but modeling long-range temporal dependencies is a challenge, while recurrent models can model sequential behavior, but they usually lack transferable feature representations. While attention mechanisms improve feature discrimination, they are not commonly combined with an explicit transfer-learning mechanism. TL-CRANN overcomes this limitation by integrating transferable CNN feature extraction, Bidirectional LSTM temporal modeling, and multi-head self-attention in an end-to-end learning framework.

#### 1.2.3 Computational efficiency for IoT deployment

Deep learning models that incorporate high-dimensional feature representations, and that follow architectures such as transformers with multiple layers, impose a significant computational overhead, preventing their deployment on IoT devices with limited resources. TL-CRANN enhances computational complexity by recursive feature elimination, hierarchical feature extraction, global average pooling and a lightweight classification head without compromise accuracy of detection.

Thus, TL-CRANN offers a single solution that combines transfer-learning, spatial feature extraction, temporal analysis of the data and adaptive attention for enhancing cross-domain IoT intrusion detection. To highlight the methodological novelty of our framework, Table 1 presents a comparison between TL-CRANN and the representative models of CNN-based, CNN–LSTM-based, attention-based, transformer- based, and transfer-learning based NID in terms of their learning paradigms, transfer-learning applicability, and major drawbacks.

**Table 1:** Methodological comparison of TL-CRANN with representative IoT intrusion detection frameworks.

Model category	Cross-domain learning	Spatial feature learning	Temporal modeling	Attention mechanism	Transfer-learning strategy	Primary limitation
CNN-based IDS	*X*	✓	*X*	*X*	*X*	Limited temporal modeling and poor cross-domain generalization.
CNN–LSTM Hybrid IDS	*X*	✓	✓	Limited/None	*X*	Dataset-specific learning with limited domain adaptation.
Attention-based IDS	*X*	Partial	Partial	✓	*X*	Improved feature weighting but limited cross-domain capability.
Transformer-based IDS	Limited	✓	✓	✓	Full model fine-tuning	High computational and memory complexity.
Transfer-learning-based IDS	✓	✓	Limited	Limited	CNN or recurrent feature transfer	Limited integration of transferable spatial, temporal, and attention learning.
TL-CRANN (proposed)	✓	✓	✓(Bi-LSTM)	✓(Multi-head self-attention)	Selective CNN layer freezing with target-domain fine-tuning	Unified framework for transferable spatial learning, temporal modeling, and adaptive attention.

### 1.3 Key contributions

The main contributions of this work are outlined as:

• We present TL-CRANN, a novel hybrid model based on transfer-learning, CNN, BiLSTM, and multi-head self-attention to detect IoT intrusion in cross-domain.
• We present a selective transfer-learning approach for heterogeneous IoT systems with scarce labeled target-domain instances that maintains transferable low-level features and adapts higher-level representations.
• We propose a hierarchical learning schema consisting of spatial feature extraction, temporal sequence modeling and attention-based feature enhancement for better representation learning of attack.
• We perform extensive experiments on CICIDS2017, TON_IoT and Bot-IoT datasets to demonstrate the cross-domain generalization of the proposed framework.
• The performance of TL-CRANN is validated by comparative and ablation experiments with representative machine learning, deep learning, transfer-learning, attention-based, and transformer-based IoT intrusion detection models.

## 2 Related work

This section reviews new directions regarding IoT intrusion detection by framing the work with the key problems in cross-domain detection, such as domain adaptation, spatial-temporal feature learning, computational efficiency, class-imbalance, and attention-based learning. We review research papers selectively to address issues that motivate the proposed TL-CRANN framework.

### 2.1 Domain adaptation for cross-domain IoT intrusion detection

However, intrusion detection in heterogeneous IoT domains is problematic since the characteristics of network traffic, the distribution of various attacks, and the feature spaces across datasets are often vastly different. Transfer-learning has become an appropriate method for migrating a model to a target-domain that has few labels. Gamlo et al. (2026) shows that pre-trained CNNs are effective in transferring the trained feature representations toward network intrusion detection and Lin et al. (2022) shows transfer-learning helps improve the generalization capability for encrypted traffic classification. Recently, adversarial trained domain adaptation approaches and methods for calculating the maximum mean difference between distributions are being studied to mitigate distribution shifts (Ma et al., 2023).

After that, TL was combined to IoT security scenarios. Many researchers employed CNN-Bi-LSTM to detect bots and malwares or classify intrusion in Industrial IoT and achieved better adaptation performances, but the transfer operation only utilizes convolutional or recurrent part independently for each, giving little instruction of selective transfer of model parameters (Nandanwar, 2023; Bosinaki et al., 2025; Wei et al., 2025). The fusion approach between transferability feature learning, temporal modeling and adaptive attention still an unsolved task, motivating the TL strategy in TL-CRANN.

### 2.2 Spatial–temporal feature learning for IoT intrusion detection

Deep learning has contributed to making tremendous progress in intrusion detection, where features can automatically be extracted from the network traffic. CNN based model can learn spatial locality across features in network flows and consistently perform better than traditional ML models (Vinayakumar et al., 2019; Khan, 2021). CNN cannot effectively model temporal long-range dependencies that are observed in changing attack behaviors. For this purpose, RNN like LSTM, GRU have been used to model attack patterns based on sequences (Alayash et al., 2026), hybrid models like CNN-LSTM are used by combining spatial feature extraction and temporal modeling.

Hybrid models have also been successfully used and extended to Bi-directional LSTM (which can model both backward and forward relations and capture more context) and has potential to improve the detection of multi-stage attacks (Zhang et al., 2023; Altunay and Albayrak, 2023). While hybrid models, although effective, usually are demonstrated on individual datasets and do not exhibit that they are able to work over different heterogeneous environments of IoT. Here, the combination of transferrable spatial-temporal learning mechanism including good modeling of temporal dimension would be expected as applied in TL-CRANN.

### 2.3 Computational efficiency for IoT intrusion detection

Efficient intrusion detection needs to be deployed on low-resource IoT device, with which contains less computation, memory and power resources. Typical CNN-based intrusion detection can provide efficient prediction time, but has limited abilities to do time-modeling. Meanwhile, traditional transformer-based intrusion detection models can carry powerful represent learning with self-attention mechanism but spend lots more computation and memory (Vaswani et al., 2017; Andresini et al., 2021).

Some previous research has been made on designing hybrid shallow architecture (with only some CNNs and RNNs together) for balance the efficiency and detection accuracy, but the deeper of these hybrid architectures is often need lots more parameters and cost (Nayak and Pawar, 2026).

It is challenging for us to get a powerful detection, efficient computation time and good performance on other different domains at the same time, that is why we design TL-CRANN in this work.

### 2.4 Class imbalance in IoT intrusion detection

Class imbalance continues to be a challenging problem in IoT intrusion detection as benign traffic and large attack families usually have much more samples than small attack families. This skew causes learning algorithms to favor the majority class, resulting in poor detection of rare yet vital attacks.

To address this issue, existing work have applied oversampling techniques such as SMOTE (Villafranca et al., 2026), under sampling approaches, cost-sensitive learning and focal loss (Dina et al., 2023; Hemalatha, 2026). Although these methods enhance the performance on minority-class detection in one single dataset, their performance tends to deteriorate when the trained models are directly used in cross-dataset IoT environments due to different attack distributions. Therefore, it is still needed to develop strong learning schemes that can guarantee a balanced detection performance for different IoT datasets, which inspires the balanced training scheme adopted in TL-CRANN.

### 2.5 Attention mechanisms for adaptive feature learning

Attention mechanisms gradually play a dominant role in enhancing feature discrimination of deep learning-based intrusion detection. Multi-head self-attention mechanisms of transformers offer better contextual representation modeling and has recently been successfully applied to network intrusion detection (Cheng, 2025; Xin, 2025). Attention based LSTM models have already been introduced and made the models learn the informative traffic sequences rather than irrelevant sequences and achieved high performances (Alabbadi and Bajaber, 2025), besides, the channel and spatial attention mechanisms improve malware detection and network traffic classification (Fan et al., 2025).

However, most attention-based intrusion detection models are mainly tested in the scope of a particular domain and performance optimization. Few research focus on performance enhancement in cross-domain cases including generalization potential for heterogenous IoT data with transferable features. Therefore, we design to introduce multi-head self-attention along with the transfer-learning in our TL-CRANN model.

### 2.6 Recent advances in hybrid and cross-domain IoT intrusion detection

Recent developments in hybrid deep learning have shown the promise of combining CNNs,RNNs with attention mechanisms for IoT intrusion detection. The TACNet and the Attention-CNN-LSTM architecture enhance the spatial–temporal feature learning by attention-guided feature representations, and two recent lightweight models featuring BiGRU, GRU, and CNN–LSTM architectures demonstrate better detection performance for Industrial IoT (IIoT) applications (Ghosh et al., 2025; Alashjaee, 2025; Ullah et al., 2023). Such studies demonstrate that hybrid attention-based models significantly enhance the quality of feature representation and the detection ability.

Despite recent advances, cross-domain generalization is one challenging task. The currently proposed hybrid, explainable and ensemble-based intrusion detection frameworks are, primarily, validated on isolated benchmark datasets and lack empirical verification on heterogeneous IoT environments (Amin et al., 2025). The importance of intrusion detection systems which simultaneously satisfy cross-domain, efficiency and explainability are also highlighted in the recently published surveys (Phalaagae et al., 2025).

### 2.7 Summary of research gaps

The literature exhibits that substantial developments have already been made on issues including transfer-learning, spatial-temporal modeling, computational efficiency, class-imbalanced handling and attention-based learning for IoT intrusion detection. Nevertheless, these issues have often been taken into consideration separately; thus, no reported works attempt to integrate transferable spatial feature extraction, bidirectional temporal modeling, adaptive attention as well as computational cost in a cross-domain ensemble. Influenced by the deficiency, this work proposed an integrated framework: TL-CRANN.

## 3 Theoretical background

### 3.1 Convolutional neural networks for feature extraction

A Convolutional Neural Network (CNNs) work by extracting nested features through multiple convolutional layers where each layer applies a set of filters *W*∈ℝ^*k*×*f*^ which are learned, to the input feature map X, with *k* being the size of the kernel and *f being* the number of input channels (Banaamah, 2022). The resulting feature map after the application of the activation is expressed function as Equation 1:


$$\begin{array}{l} Z_{l}\;=\;\sigma \left(W_{l}^{*}X+b_{l}\right) \end{array}$$ (1)

here convolution operation is denoted by ^*^ and *b* is the bias term. Max-pooling layers then reduce the spatial size while maintaining the prominent features. In the TL-CRANN structure, 1D CNN layers take the sequence of network flow feature vectors as input where each flow is considered as a temporal channel and spatial correlations at a local level between adjacent feature dimensions are being extracted.

### 3.2 Long short-term memory networks

LSTM networks solve the issue of vanishing gradients in standard RNNs by using gating mechanisms in their memory cells (Noh, 2021).

At time step *t*, LSTM calculates the hidden state *h*_*t*_ and cell state *c*_*t*_ as shown in Equations 2–6:


$$\begin{array}{l} f_{t}=\sigma \left(W_{f}\cdot \left[h_{t-1},x_{t}\right]+b_{f}\right) \end{array}$$ (2)

$$\begin{array}{l} i_{t}=\sigma \left(W_{i}\cdot \left[h_{t-1},x_{t}\right]+b_{i}\right) \end{array}$$ (3)

$$\begin{array}{l} o_{t}=\sigma \left(W_{o}\cdot \left[h_{t-1},x_{t}\right]+b_{o}\right) \end{array}$$ (4)

$$\begin{array}{l} c_{t}=f_{t}⊙c_{t-1}+i_{t}⊙\tanh\left(W_{c}\cdot \left[h_{t-1},x_{t}\right]+b_{c}\right) \end{array}$$ (5)

$$\begin{array}{l} h_{t}=o_{t}⊙\tanh\left(c_{t}\right) \end{array}$$ (6)

here *f*_*t*_, *i*_*t*_ and *o*_*t*_ are the forget, input, and output gates respectively, σ is the sigmoid function, and ⊙ means element-wise multiplication. LSTM's selective memory capability is what makes it highly effective in modeling sequential behaviors in network traffic like multi-stage attacks and slow-rate intrusion patterns.

### 3.3 Multi-head self attention mechanism

The self-attention mechanism computes a weighted representation of a sequence by attending all positions simultaneously. This mechanism works below: from the LSTM output sequence H, three main matrices are selected, the query Q, key K, and value V. Attention densifies the relationships between positions in a sequence using scaled dot-product as a measure (Umer et al., 2025). Then, for Q K V matrices, scaled dot-product attention is defined in Equation 7:


$$\begin{array}{l} Attention\left(Q,K,V\right)=softmax\left(\frac{QK^{T}}{\sqrt{d_{k}}}\right)V \end{array}$$ (7)

where *d*_*k*_ stands for the key dimensionality. Multi-head attention generalizes the idea by first linearly projecting Q K V into *h* different as subspaces defined in Equation 8:


$$\begin{array}{l} MultiHead\left(Q,K,V\right)=Concat\left(head_{1},\ldots ,head_{h}\right)W^{O} \end{array}$$ (8)

where $head_{i}=Attention\left(QW_{i}^{Q},KW_{i}^{K},VW_{i}^{V}\right)$. In TL-CRANN, multi-head attention is carried out on the LSTM hidden state sequence to generate asynchronous recognition assumed that it produces a context-aware representation which are characteristic of attack behavior.

### 3.4 Transfer-learning via fine-tuning

Transfer-learning (Harahsheh et al., 2024) uses the training experience on a source-domain *D*_*S*_ to help improve the performance on a target-domain *D*_*T*_, even when *D*_*T*_ and *D*_*S*_ are different with their feature spaces or label distributions. In the fine-tuning approach, a pre-trained model θ_*S*_ is changed either partially or fully by using the labeled data from *D*_*T*_ as defined in Equation 9:


$$\begin{array}{l} \theta_{T}=\theta_{S}-\eta \nabla L\left(D_{T};\theta_{S}\right) \end{array}$$ (9)

here η stands for the learning rate and *L* is the loss function specific to a task. In TL-CRANN, the CNN encoder is first trained on the CICIDS 2017 dataset and then fine-tuned with smaller learning rates on the domains of TON_IoT and Bot-IoT, which retains the feature representations of low-level while changing the classifiers of high-level that are related to the semantics of the target-domain attacks.

## 4 Dataset description and pre-processing

### 4.1 Dataset selection rationale

In this study, three representative IoT security datasets (Table 2) are used to test the performance of TL-CRANN under different attack scenarios and network environments. The criteria for choosing include:

**Table 2:** Summary of datasets used in TL-CRANN evaluation.

Dataset	Records	Features	Attack types	Benign %	Year
CICIDS 2017	2,830,743	80	14 categories (13 attack subtypes +Benign)	82.02	2017
TON IoT	5,351,757	44	9 categories	22.39	2020
Bot-IoT	~3,668,013^*^	46	5 categories	0.01	2020

^*^Curated version used; full dataset contains 73M+ records.

- a. recentness (2017–2020),
- b. focus on IoT,
- c. variety of attack types,
- d. publicly accessible to ensure reproducibility,
- e. large scale enough to allow deep learning training.

The datasets CICIDS 2017, TON_IoT 2020, and Bot-IoT 2020 together depict various IoT settings like smart homes, industrial IoT, and cloud-based architectures.

### 4.2 CICIDS 2017

Among all the public datasets used for network intrusion detection system research, one of the most popular one is the Canadian Institute for Cybersecurity Intrusion Detection Evaluation Dataset 2017(CICIDS 2017). The dataset was created in a practical lab setup using CICFlowMeter and contains 2,830,743 flow records collected in 5 days (Monday to Friday). Each flow record has 80 features representing network flows and consists of eight broad attack families (Brute Force (SSH-Patator, FTP-Patator), DoS/DDoS (Hulk, Slowloris, Slowhttptest, GoldenEye), Heartbleed, Web Attacks [XSS, SQL Injection, Brute Force), Infiltration, Botnet (ARES)]. For the classification task, these broad families are disaggregated into their 13 constituent fine-grained attack subtypes (e.g., DoS Hulk, DoS GoldenEye, DoS Slowloris, and DoS Slowhttptest are treated as four separate subtypes of the DoS/DDoS family), so the classification head distinguishes 13 attack subtypes plus the Benign class (14 classes total), consistent with the per-class results reported in Table 3. There is a major imbalance on classes, and benign traffic covers about 82% of all the data records.

**Table 3:** Per-attack-class detection performance of TL-CRANN on CICIDS 2017.

Attack category	Precision (%)	Recall (%)	F1-score (%)	FPR (%)	Support
Benign	99.12	98.94	99.03	0.41	463,920
DoS Hulk	98.74	98.53	98.63	0.84	115,442
PortScan	98.91	98.72	98.81	0.63	158,930
DDoS	98.63	98.41	98.52	0.73	41,835
DoS GoldenEye	97.84	97.61	97.72	1.27	5,050
FTP-Patator	98.32	98.11	98.21	1.04	3,970
SSH-Patator	98.14	97.93	98.03	1.13	3,228
DoS Slowloris	97.41	97.13	97.27	1.54	2,735
DoS Slowhttptest	96.83	96.54	96.68	1.81	2,321
Botnet ARES	97.72	97.43	97.57	1.34	1,966
Web attack XSS	95.63	95.31	95.47	2.41	652
Web attack brute force	95.41	95.12	95.26	2.54	1,307
Web attack SQLi	93.74	93.41	93.57	3.12	21
Heartbleed	99.41	99.18	99.29	0.21	11
Macro average	98.14	97.93	98.03	1.21	801,388

### 4.3 TON_IoT

The TON_IoT dataset was created by the UNSW Canberra Cyber Range Lab for the purpose of researches related to security in IoT and IIoT. It consists of network and system telemetry recorded on a simulated Cyber Range testbed that is equipped with smart home appliances, weather station sensors, and industrial Modbus controllers. The dataset consists of 5,351,757 records and has 44 network features and nine types of attack: Scanning, DoS, DDoS, Ransomware, Backdoor, Injection, XSS, Password and MITM attacks. TON_IoT is very useful for assessing cross-domain transfer-learning since its feature distribution is quite different from the one presented by CICIDS 2017.

### 4.4 Bot-IoT

The Bot-IoT dataset was created at UNSW Canberra Cyber Range to include realistic IoT botnet attacks like DDoS, DoS, OS Fingerprinting, Service Discovery, Data Exfiltration and Keylogging. These attacks are implemented using IoT botnet scripts to attack IoT devices such as smart thermostat, smart refrigerator, and weather monitoring systems. The complete dataset contains over 73 million records; in this study we used 5 classes, 5 features version, extended to full 46 features. There is a huge imbalance in the Bot-IoT dataset, since the number of DDoS attacks reached 94% out of all attacks.

### 4.5 Data pre-processing pipeline

A standard six stage pre-processing pipeline adopted for all the three datasets (Figure 1) for uniform processing and optimum model performance. To prevent data leakage, the stratified train/validation/test split (70/10/20%, Section 6.1) is always performed first, before Stages 1 and 5; the RFE feature selector in Stage 1 is fit exclusively on the training partition and the resulting feature subset is then applied unchanged to the validation and test partitions, and the SMOTE oversampling and under-sampling in Stage 5 are applied only to the training partition, leaving the validation and test partitions in their original, naturally imbalanced class distribution so that reported performance reflects real-world class proportions.

**Figure 1:**
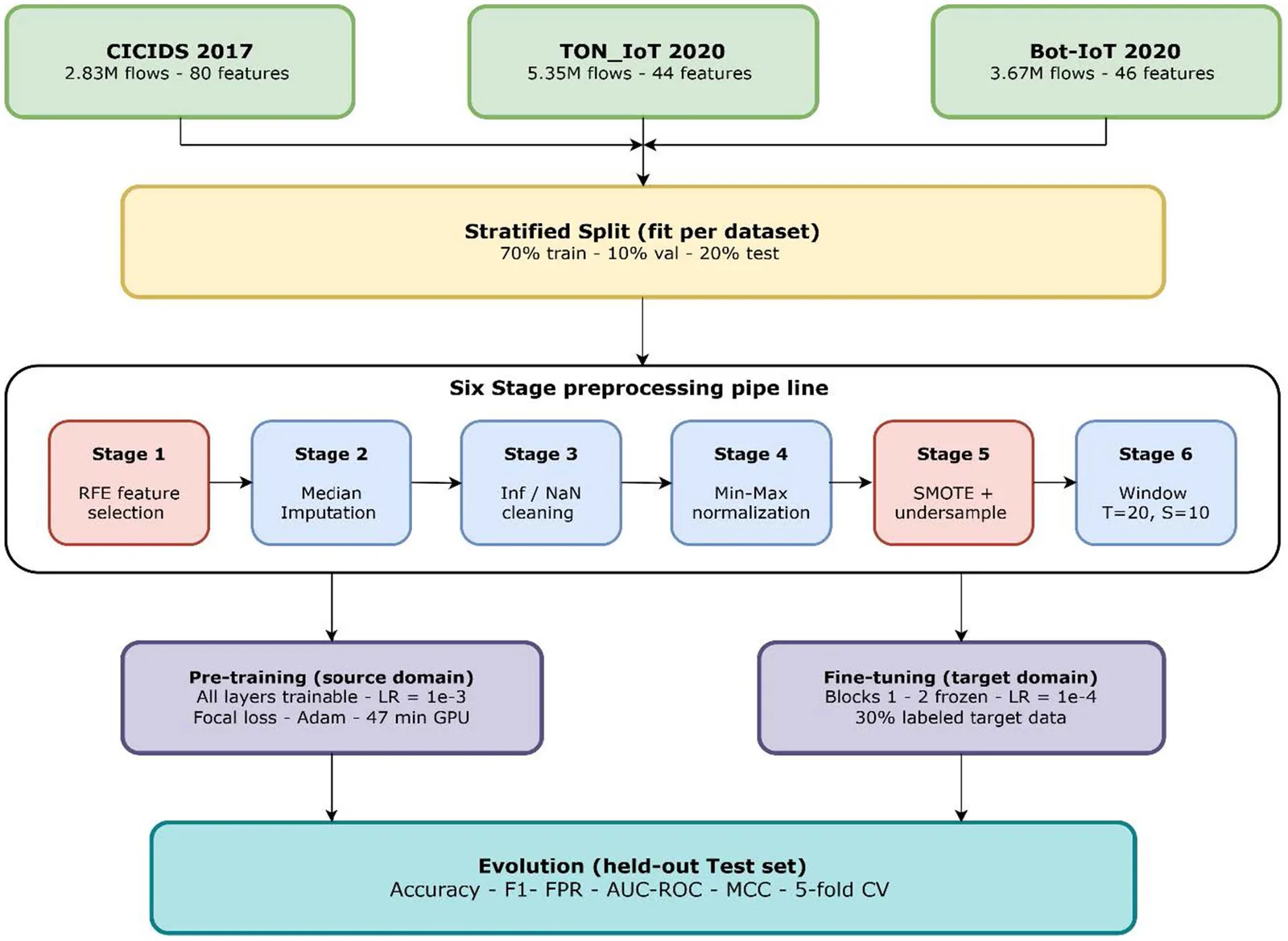
Six-stage pre-processing pipeline. RFE and SMOTE are fit exclusively on the training partition after stratified splitting.

Stage 1–Feature selection: random forest based recursive feature elimination (RFE) reduce the feature dimensions to 40 for CICIDS 2017, 30 for TON_IoT and 35 for Bot-IoT datasets, while low-variance features (variance < 0.01) were eliminated. The Random Forest ranker underlying RFE, and the resulting selected feature subset, are both fit only on the training partition of each dataset; the same subset of features is then simply subset (not re-fit) from the validation and test partitions.

Stage 2–Handling missing values: features with more than 5% missing values were discarded. Remaining missing values in the datasets were imputed using the median of their respective features to preserve the data distribution and mitigate the effects of skewness and outliers.

Stage 3–Infinities and NaN values: the resulting inf values from flow duration division-by-zero were replaced by the max possible finite value in the same column and NaN values obtained post imputation are substituted by zeros.

Stage 4–Normalization: the features are scaled using min-max normalization [0, 1] method over the entire training dataset and used on test sets too (to avoid data leak).

Stage 5–Handling class imbalance: over sampling method SMOTE applied over the minority class (attacks) until 15% count of the majority class (benign) was achieved and under sampling is used on majority class to get a 3:1 ratio to the minority class. Both SMOTE oversampling and under-sampling are applied strictly to the training partition after the train/validation/test split; the validation and test partitions are never resampled and retain their original class proportions, so reported metrics are computed on realistically imbalanced data.

Stage 6–Building sequences: feature vectors are then transformed into sequences by sliding window approach of size *T* = 20 and stride *S* = 10, which resulted in [*x*_1_, *x*_2_, …, *x*_*T*_] as input sequences for the sequential LSTM.

## 5 Proposed TL-CRANN architecture

### 5.1 Architectural overview

The proposed TL-CRANN (Figure 2) model consists of a five-level deep learning pipeline: (1) Transfer-learning-Based CNN Encoder, (2) Temporal Context Reduction, (3) Bidirectional LSTM Temporal Modeler, (4) Multi-Head Self-Attention Layer, and (5) Classification Head. An illustration of the whole model architecture is given in Figure 2. The design approach of TL-CRANN is built on three purposes: (i) transferable low-level feature representation through pre-trained CNN weights, (ii) effectively modeling sequential attack patterns through stacked Bi-LSTM layers and (iii) discriminative temporal focus through multi-head attention, enabling the model to selectively attend to attack relevant timesteps while suppressing benign background traffic. Table 4 presents the complete layer-wise configuration and output shapes of TL-CRANN.

**Figure 2:**
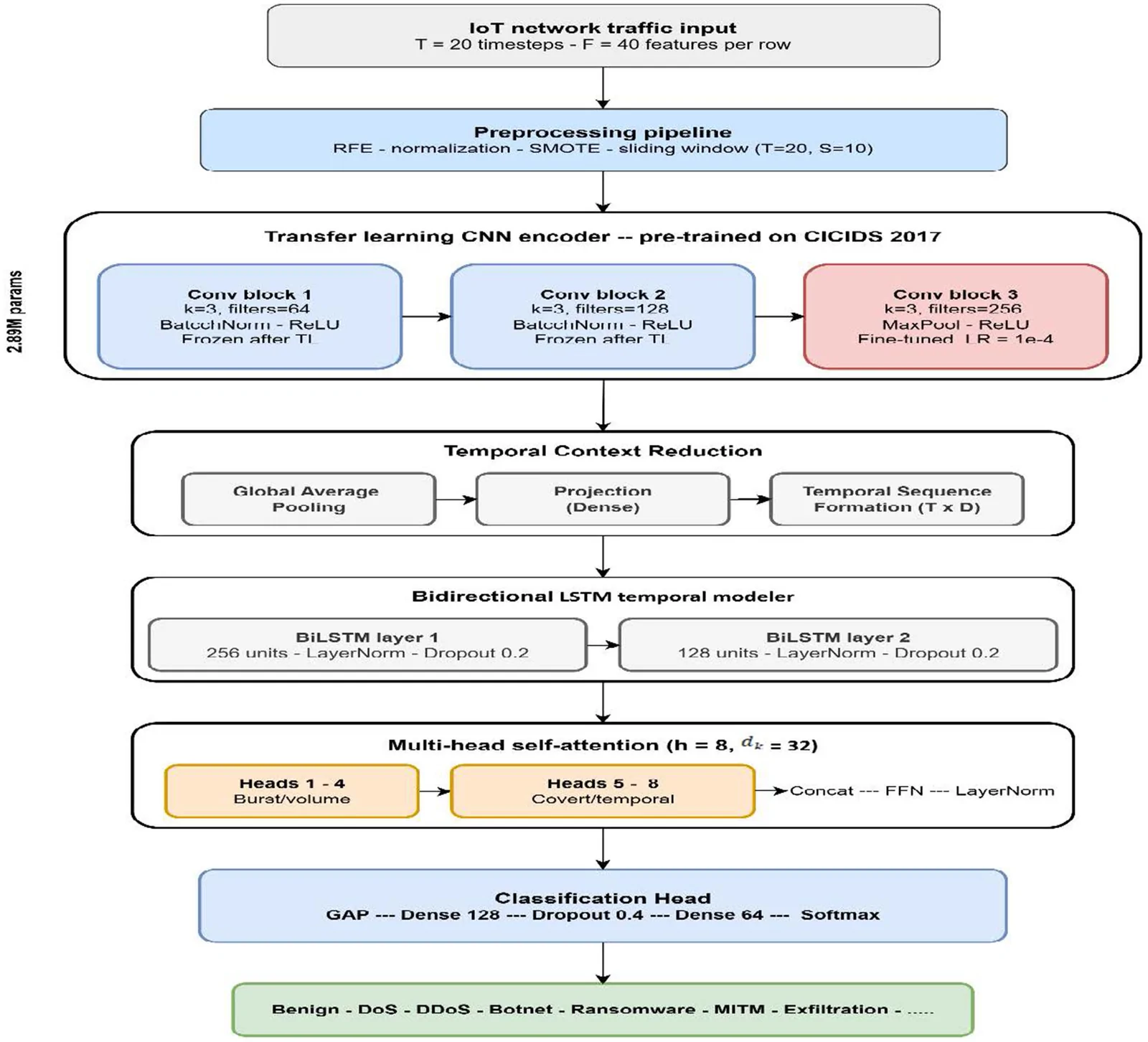
TL-CRANN system architecture showing the five-stage pipeline: transfer-learning CNN encoder, max pooling, Bi-LSTM temporal modeler, multi-head self-attention, and classification head.

**Table 4:** TL-CRANN layer configuration and output shapes.

Module	Layer type	Parameters	Output shape
Input	Sequence Input	T = 20, F = 40 features	(Batch, 20, 40)
CNN Encoder (TL)	Conv1D (k = 3, f = 64) + BN + ReLU	Pre-trained on CICIDS	(Batch, 20, 64)
CNN Encoder (TL)	Conv1D (k = 3, f = 128) + BN + ReLU	Fine-tuned	(Batch, 20, 128)
CNN Encoder (TL)	Conv1D (k = 3, f = 256) + BN + ReLU	Fine-tuned	(Batch, 20, 256)
Pooling	MaxPool1D (k = 2)	Stride = 1	(Batch, 20, 256)
Bi-LSTM Layer 1	Bidirectional LSTM	Units = 256 (128 each dir.)	(Batch, 20, 512)
Bi-LSTM Layer 2	Bidirectional LSTM	Units = 128 (64 each dir.)	(Batch, 20, 256)
Attention	Multi-head self-attention	h = 8 heads, d_k = 32	(Batch, 20, 256)
Aggregation	Global average pooling	—	(Batch, 256)
FC Layer 1	Dense + BatchNorm + Dropout(0.4)	Units = 128	(Batch, 128)
FC Layer 2	Dense + BatchNorm + Dropout(0.3)	Units = 64	(Batch, 64)
Output	Dense + Softmax	*N* classes	(Batch, *N*)

### 5.2 Transfer-learning CNN encoder

TL-CRANN's CNN encoder is composed of three stacked 1D conv blocks, each formed by a 1D Conv layer with ReLU, Batch Normalization and possibly Max pooling. The encoder is pre-trained on the whole CICIDS 2017 dataset, in a supervised manner (cross-entropy loss) for 14-class classification. Through pre-training, it creates a learned representation space in which network flow features are mapped into distinctive spatial subspace based on their type of attack. Because RFE selects a different number of input features per dataset (40 for CICIDS 2017, 30 for TON_IoT, 35 for Bot-IoT; Section 4.5, Stage 1), a lightweight, dataset-specific linear input-adapter layer (a 1x1 convolution/fully connected projection, not shared and not frozen) first maps each dataset's post-RFE feature vector onto the common 40-dimensional channel width expected by the pre-trained first convolutional block, padding or projecting as needed. This adapter is trained from scratch for every target domain, while the frozen convolutional blocks downstream of it always operate on inputs of the fixed dimensionality, they were pre-trained on, so the transferred low-level filters remain valid regardless of the raw feature count of the source dataset.

While performing cross-domain transfer to TON_IoT and Bot-IoT datasets, the first convolutional blocks (low-level feature extractors) are frozen, while the last convolutional block and the whole upper layers are fine-tuned with a learning rate of 1e-4 (10 times smaller than the pre-training learning rate 1e-3). This partial freezing technique enables to preserve general network flow features (like packet rate, byte count distributions etc) and fine-tune specific attack signatures. The parameter budget of the frozen CNN encoder is 247,808 (31.2% of the total parameter budget of the model). Figure 3 illustrates the pre-training and cross-domain fine-tuning strategy.

**Figure 3:**
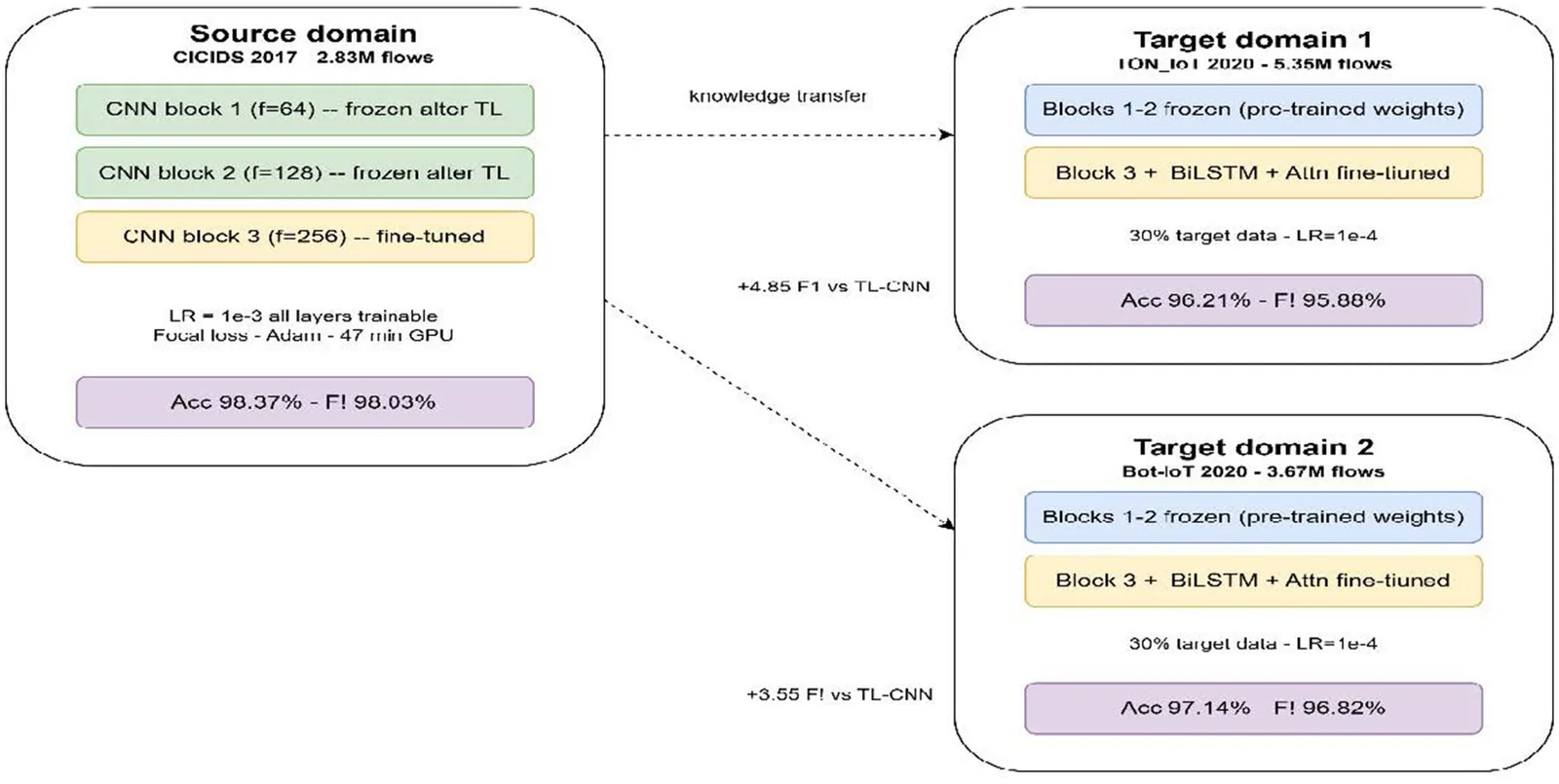
Transfer-learning strategy: Phase 1 pre-training on CICIDS 2017 (source-domain) and Phase 2 selective fine-tuning on TON_IoT and Bot-IoT target-domains with frozen low-level CNN blocks.

### 5.3 Bidirectional LSTM temporal modeler

The encoded features extracted by the CNN encoder are then passed to a two-layer stack of Bi-LSTM. Bidirectional propagation allows the network to use both the previous and future flow history in the current window to identify attack signature which propagates across a few consecutive flow, e.g., Slow-rate DoS and multi-stage exfiltration. First layer of Bi-LSTM outputs a 512-dimension hidden sequence (256 for each direction), which extracts the detailed sequential dynamics. Second layer of Bi-LSTM outputs a 256-dimension sequence, imposing a bottleneck which is enforced for generating a discriminative yet compact representation.

Recurrent dropout with *p* = 0.2 is added to both Bi-LSTM cells on the recurrent kernel for better regularization while not breaking the temporal state. Layer Normalization is applied in between the two layers of Bi-LSTM for more robust training.

### 5.4 Multi head attention layer

The Bi-LSTM output hidden state sequence $H\;=\;\left\{h_{1},h_{2},\ldots ,h_{T}\right\}\in {\mathbb{R}}^{T\times 256}$ is fed into the multi-head self-attention layer, which calculates attentions across all timesteps at once. Eight attention heads (*h* = 8) project the 256-d vectors to 32-d (*d*_*k*_ = *d*_*v*_ = 32) subspaces to get query, key and value. Then the resultant context matrix *C* = *MultiHead*(*H, H, H*)∈ℝ^*T*×256^ is fed into a position-wise feedforward network (FFN) with hidden dimension 512 with a residual connection, as defined in Equation 10:


$$\begin{array}{l} Z=Layer\;Norm\left(C+FFN\left(C\right)\right) \end{array}$$ (10)

With the multi-head self-attention layer, TL-CRANN is able to focus on multiple scales and feature dimensions in parallel, some heads focusing on short duration attacks that are indicative of DDoS amplification while others can focus on prolonged, low-rate behavior that might indicate reconnaissance or exfiltration of data. The attention weights visualized below in Section VII-D provide an illustration of this capability.

### 5.5 Classification head

The attention output tensor *Z*∈ℝ^*T*×256^ is reduced to an invariable length of 256-dimensional feature vector using Global Average Pooling (GAP) along the time axis so that the representation is independent of the sequence length and available to use in downstream classification. From this, an intermediary representation is produced in passing two fully connected (FC) layers (with 128 and 64 units, respectively, as the number of neurons), each of which has Batch Normalization (BN) applied to it, then dropout at two rates (0.4 and 0.3), and finally a Softmax layer generating *N* category probability estimates corresponding to the classification task as:

• *N* = 14 (13 attack subtypes + Benign) in CICIDS 2017.
• *N* = 10 (9 attack types + Benign) in TON_IoT 2020.
• *N* = 6(5 attack types + Benign) in Bot-IoT 2020.

Using different progressively increasing dropout rates (0.4 and 0.3) across the two FCs encourages a form of structured regularity where strongest regularization occurs close to the attention bottleneck, thus avoiding interdependency between high level features whilst retaining attack class-specific information.

### 5.6 Training strategy, hyperparameter optimization, and reproducibility

The proposed TL-CRANN model was trained by Adam with the initial learning rate being set to 1 × 10^−3^ in source-domain pre-training and to 1 × 10^−4^ in target-domain fine-tuning, respectively. In order to balance the class, the Focal Loss function (Equation 11) was utilized as the optimization target,


$$\begin{array}{l} FL\left(p_{t}\right)=-\alpha_{t}{\left(1-p_{t}\right)}^{\gamma }\log\left(p_{t}\right) \end{array}$$ (11)

where *p*_*t*_ represents the estimated probability of the true class, α_*t*_ is the class-balancing term, and γ re-weights the importance of easy/hard training examples.

The hyperparameters were jointly tuned on the validation set by monitoring the macro F1 score, while at the same time ensuring convergence to a stable solution. The complete training setting such as the optimizer, learning rates, batch size, number of Bi-LSTM hidden units, number of attention heads, dropout rate and early stopping condition is listed in Table 5. We have applied Early stopping and ReduceLROnPlateau to help with the convergence and overfitting.

**Table 5:** Hyperparameter configuration of the proposed TL-CRANN framework.

Category	Hyperparameter	Value
Optimizer	Adam	Default
Learning rate	Source-domain pre-training	1 × 10^−3^
Learning rate	Target-domain fine-tuning	1 × 10^−4^
Batch size	Training batch size	512
Epochs	Maximum training epochs	100
Learning rate scheduler	ReduceLROnPlateau	Factor = 0.5
Early stopping	Patience	15 epochs
CNN	Kernel size	3 × 3
Bi-LSTM	Hidden units	256/128
Multi-head attention	Number of heads	8
Dropout	CNN/dense layers	0.4/0.3

For the sake of reproducibility, the code of our framework was written in Python with TensorFlow/Keras and Scikit-learn, and the experiments were conducted on an NVIDIA A100 GPU (40 GB) with 64 GB system memory. In transfer-learning, freezing low-level convolutional layers and fine-tuning higher-level CNN layers, Bidirectional LSTM, multi-head self-attention, and classification layers, to fine-tune the model on the target-domain.

## 6 Experimental setup

### 6.1 Evaluation protocol

Stratified sampling is employed to split each dataset into 70% training, 10% validation, and 20% testing to maintain the original class distribution in all subsets. The same data division method was used for all the datasets and competing models for a fair performance evaluation and results reproducibility.

TL-CRANN is pre-trained on the CICIDS2017 dataset and then fine-tuned with 30% of the labeled target-domain data from either TON_IoT or Bot-IoT to assess supervised cross-domain transfer-learning. The rescinding 70% of the data in the target-domain was kept for testing only. The evaluation procedure is aligned with real applications practical IoT deployments in which only a small amount of labeled target-domain traffic is for model adaptation and an independent test set is preserved for unbiased performance evaluation.

### 6.2 Performance metrics

The model performance is evaluated by the following metrics, calculated per class and then macro-averaged (i.e., the unweighted arithmetic mean of the per-class scores, giving every class-majority or minority-equal influence on the reported value). This unweighted macro-averaging, rather than support-weighted averaging, is used throughout the paper precisely because class imbalance is central to our evaluation: a support-weighted average would be dominated by the Benign and high-frequency attack classes and could mask poor detection of rare attack types. The evaluation metrics are defined in Equations 12–17:


$$\begin{array}{l} Accuracy=\frac{TP+TN}{TP+TN+FP+FN} \end{array}$$ (12)

$$\begin{array}{l} Precision=\frac{TP}{TP+FP} \end{array}$$ (13)

$$\begin{array}{l} Recall=\frac{TP}{TP+FN} \end{array}$$ (14)

$$\begin{array}{l} F1=\frac{2\times Precision\times Recall}{Precision+Recall} \end{array}$$ (15)

$$\begin{array}{l} FPR=\frac{FP}{FP+TN} \end{array}$$ (16)

$$\begin{array}{l} MCC=\frac{TP\times TN-FP\times FN}{\sqrt{\left(TP+FP\right)\left(TP+FN\right)\left(TN+FP\right)\left(TN+FN\right)}} \end{array}$$ (17)

### 6.3 Baseline models

TL-CRANN is compared with following state-of-the-art models, which all are re-implemented using the same pre-processing pipeline and computational environment:

• Random Forest (RF): 200 trees, max depth = 30
• Support vector machine (SVM): RBF kernel, C = 10, = 0.01
• Deep Neural Network (DNN): five fully connected layers
• 1D-CNN: 3 convolution blocks, with same hyperparameters as the CNN encoder in TL-CRANN
• LSTM: 2-layer LSTM, 256 units for each layer
• CNN-LSTM: sequentially stacking CNN and LSTM, no attention applied
• Bi-LSTM: 2-layer bidirectional LSTM, no attention applied
• TL-CNN: transfer-learning model with CNN only, no LSTM and attention applied On the source domain (CICIDS 2017), where there is no prior domain to transfer from, TL-CNN is trained from scratch exactly like the other baselines (i.e., it reduces to a plain CNN classifier); the “transfer-learning” behavior specific to TL-CNN, namely pre-training on CICIDS 2017 followed by partial-freezing fine-tuning, is only exercised when it is evaluated on the target domains, TON_IoT and Bot-IoT.
• BERT-IDS: transformer-based IDS designed for network data
• Attention-LSTM: LSTM model with single-head attention applied, no CNN and no TL applied

## 7 Results and analysis

The TL-CRANN has been assessed using comprehensive, and equally weighted, evaluation on all three datasets: CICIDS 2017, TON_IoT 2020 and Bot-IoT 2020. For each of these datasets, we include: (1) complete model comparison against 10 baselines, (2) per class detection performance and (3) dataset-specific analytical discussion of equal depth. Finally, we provide cross-dataset discussions on ablation study, interpretability with multi-head attention, computational complexity and statistical tests. The headline comparison tables for each dataset report macro-averaged results on a single held-out test partition (Section 6.1); the 5-fold stratified cross-validation is used specifically for the statistical robustness analysis in Section 7.10 (means, standard deviations, and 95% confidence intervals).

### 7.1 Evaluation on CICIDS 2017 dataset

CICIDS 2017 is used as the pre-training source-domain for TL-CRANN and contains the largest span of attack categories among the three datasets (fourteen categories over five capture days). All models were trained end-to-end on the 70/10/20 stratified split, and were tested on the withheld test set of 566,838 instances. TL-CRANN was pre-trained on CICIDS 2017 with a random initialization of all weights; none were loaded from a pre-existing source for this set.

As detailed in Table 6, TL-CRANN surpasses the competitive models on the CICIDS 2017 dataset with an impressive accuracy of 98.37%, outperforming BERT-IDS (97.43%) and TL-CNN (97.12%) respectively. With the lowest FPR of 0.84% the model brings a decrease in unwanted alarms of 35.9 and 70.3% compared to BERT-IDS and Random Forest model, respectively. Also, the obtained value of MCC score is 0.9791 which shows that the performance of TL-CRANN remains strong even in the presence of class imbalance and AUC-ROC is 0.9974 which depicts a great separability between the benign and attack classes.

**Table 6:** Overall performance comparison on CICIDS 2017 (Multi-Class, Macro-Averaged).

Model	Accuracy (%)	Precision (%)	Recall (%)	F1-score (%)	FPR (%)	AUC-ROC	MCC
Random Forest	95.14	94.27	93.61	93.93	2.83	0.9784	0.9312
SVM	93.72	92.84	92.17	92.5	3.64	0.9671	0.9121
DNN	95.43	94.71	94.13	94.41	2.61	0.9812	0.9384
1D-CNN	95.87	95.14	94.83	94.98	2.24	0.9843	0.9451
LSTM	95.61	94.92	94.57	94.74	2.41	0.9831	0.9421
CNN-LSTM	96.34	95.72	95.41	95.56	1.93	0.9871	0.9523
Bi-LSTM	96.71	96.18	95.84	96.01	1.74	0.9884	0.9561
TL-CNN	97.12	96.83	96.54	96.68	1.47	0.9913	0.9621
BERT-IDS	97.43	97.11	96.82	96.96	1.31	0.9931	0.9662
Attention-LSTM	97.21	96.94	96.63	96.78	1.42	0.9922	0.9641
TL-CRANN (Ours)	98.37	98.14	97.93	98.03	0.84	0.9974	0.9791

#### 7.1.1 Confusion matrix analysis—CICIDS 2017

Figure 4 shows the Normalized Confusion Matrix of the classification outcomes in the all 14 traffic types (Table 3); all detection rates are above 93%. Only a small number of packets between DoS Slowloris and DoS Slowhttptest have minor misclassification because they have similar traffic flow characteristics. Web Attack SQLi achieves the worst score on F1-score (93.57%), since the sample size is rather small, and its traffic pattern resembles benign web traffic. The true negative rate (TNR) of the Benign class is high (99.03%), and the false positive rate (FPR) is low (0.84%); in this regard, false alarms would be very few. For heavy attacks like PortScan (98.81%), DoS Hulk (98.63%), and DDoS (98.52%), the detection rates are great. Despite its rarity, Heartbleed was detected with 99.29% accuracy, while all attack achieved F1-scores above 93.5%.

**Figure 4:**
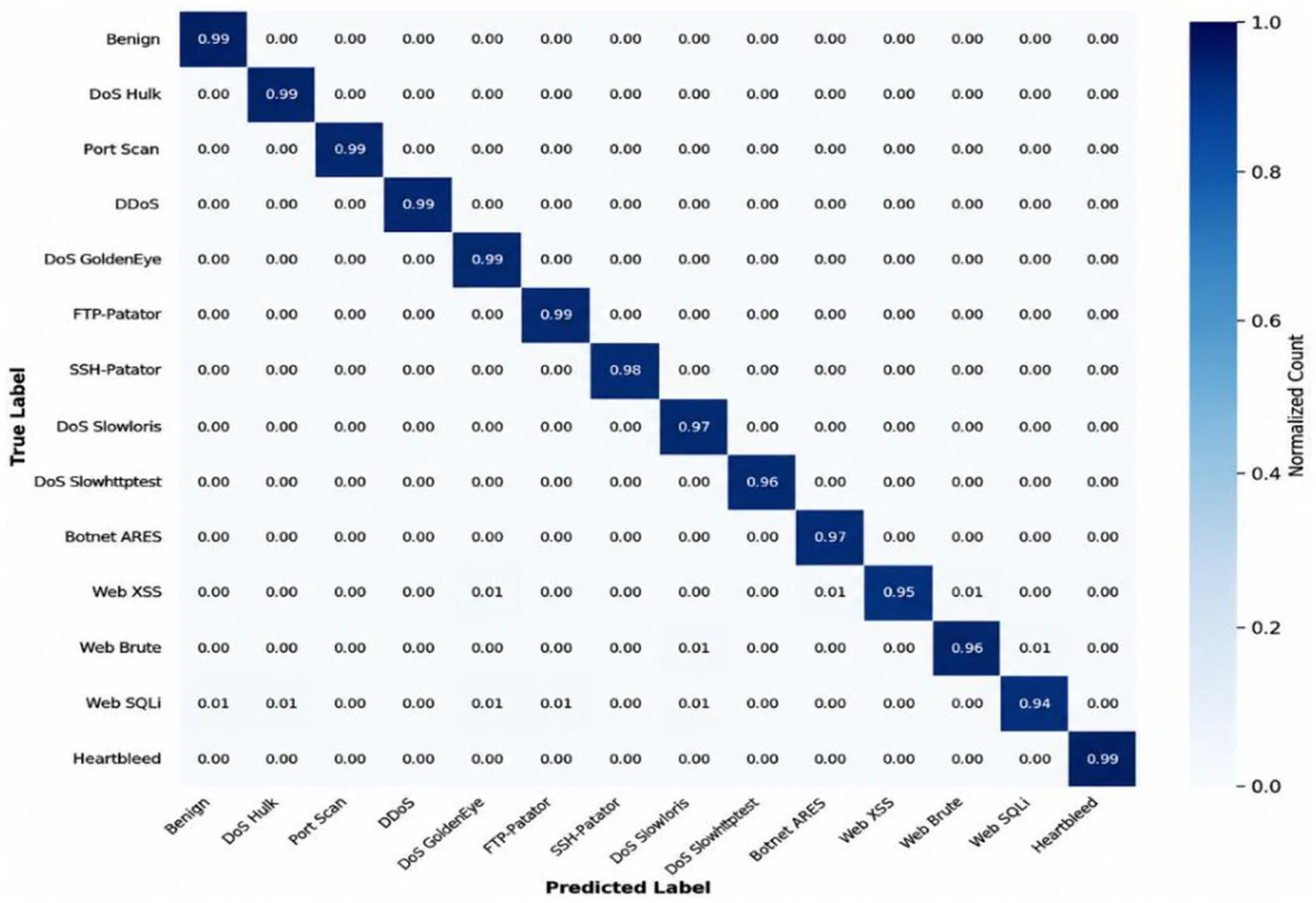
Normalized confusion matrix of TL-CRANN on the CICIDS 2017 test set (14 attack categories).

### 7.2 Evaluation on TON_IoT 2020 dataset

The TON_IoT 2020 dataset, simulated in a custom-built Cyber Range equipped with heterogeneous IoT and IIoT devices such as smart thermostats, weather sensors and Modbus industrial controllers, presents a challenging cross-domain transfer scenario due to its unique 44-feature flow scheme and presence of attack categories that were not present in CICIDS 2017 (Ransomware, MITM, Password attacks). TL-CRANN is fine-tuned using 30% of the TON_IoT training data (first two blocks of the CNN encoder are frozen), and is evaluated on the remaining 70% portion of the test data. All other baselines are trained from scratch on the same 30% data partition used for fine-tuning for comparison purposes.

The Table 7 indicates the better cross-domain generalizability for TL-CRANN under TON_IoT. Traditional classifiers, such as Random Forest, CNN-LSTM, and 1D-CNNs, show remarkable declines when faced with the domain drift, whereas models benefiting from transfer-learning exhibit enhanced adaptability. TL-CRANN achieves promising experimental results with 96.21% accuracy, 95.88% macro F1-score and only 1.27% FPR, the former exceeds TL-CNN and BERT-IDS by more than 4.8 percentage points in terms of F1-score. Moreover, 0.9541 of MCC confirms its performance to classify with extreme class imbalance well; Therefore, the combination of transfer-learning, attention mechanisms, and temporal sequence modeling helps achieving cross-domain intrusion detection.

**Table 7:** Overall performance comparison on TON_IoT 2020 (multi-class, macro-averaged).

Model	Accuracy (%)	Precision (%)	Recall (%)	F1-score (%)	FPR (%)	AUC-ROC	MCC
Random Forest	85.73	84.92	83.61	84.26	4.73	0.9421	0.8284
SVM	82.14	81.37	80.84	81.1	6.14	0.9183	0.7971
DNN	86.42	85.71	84.93	85.31	4.47	0.9483	0.8392
1D-CNN	84.17	83.44	82.73	83.08	5.21	0.9347	0.8163
LSTM	85.91	85.14	84.52	84.82	4.63	0.9437	0.8341
CNN-LSTM	87.14	86.53	85.92	86.22	4.13	0.9534	0.8473
Bi-LSTM	87.83	87.12	86.54	86.83	3.91	0.9571	0.8541
TL-CNN	91.74	91.23	90.84	91.03	2.63	0.9741	0.9001
BERT-IDS	91.12	90.74	90.31	90.52	2.84	0.9712	0.8941
Attention-LSTM	89.43	88.82	88.34	88.58	3.42	0.9621	0.8743
TL-CRANN (Ours)	96.21	96.03	95.74	95.88	1.27	0.9951	0.9541

#### 7.2.1 Confusion matrix analysis—TON_IoT 2020

The confusion matrix details (Figure 5) how TL-CRANN can discriminate classes (Table 8). Classes like DDoS (97.27%), Scanning (96.98%), DoS (96.68%), Ransomware (94.57%), Password (93.62%) and MITM (92.93%), showed excellent classification, although there was slight confusion between Scanning traffic and MITM. As for class Benign, the classifier produced a satisfactory F1-score of 97.73 and 0.73% class-level FPR (effectively limiting false alarms). On average, all the classes secured a F1-score exceeding 92.9%, reducing the overall FPR by 45.8% as compared to the BERT-IDS, which clearly indicates that TL-CRANN can perform well in disparate IoT environments.

**Figure 5:**
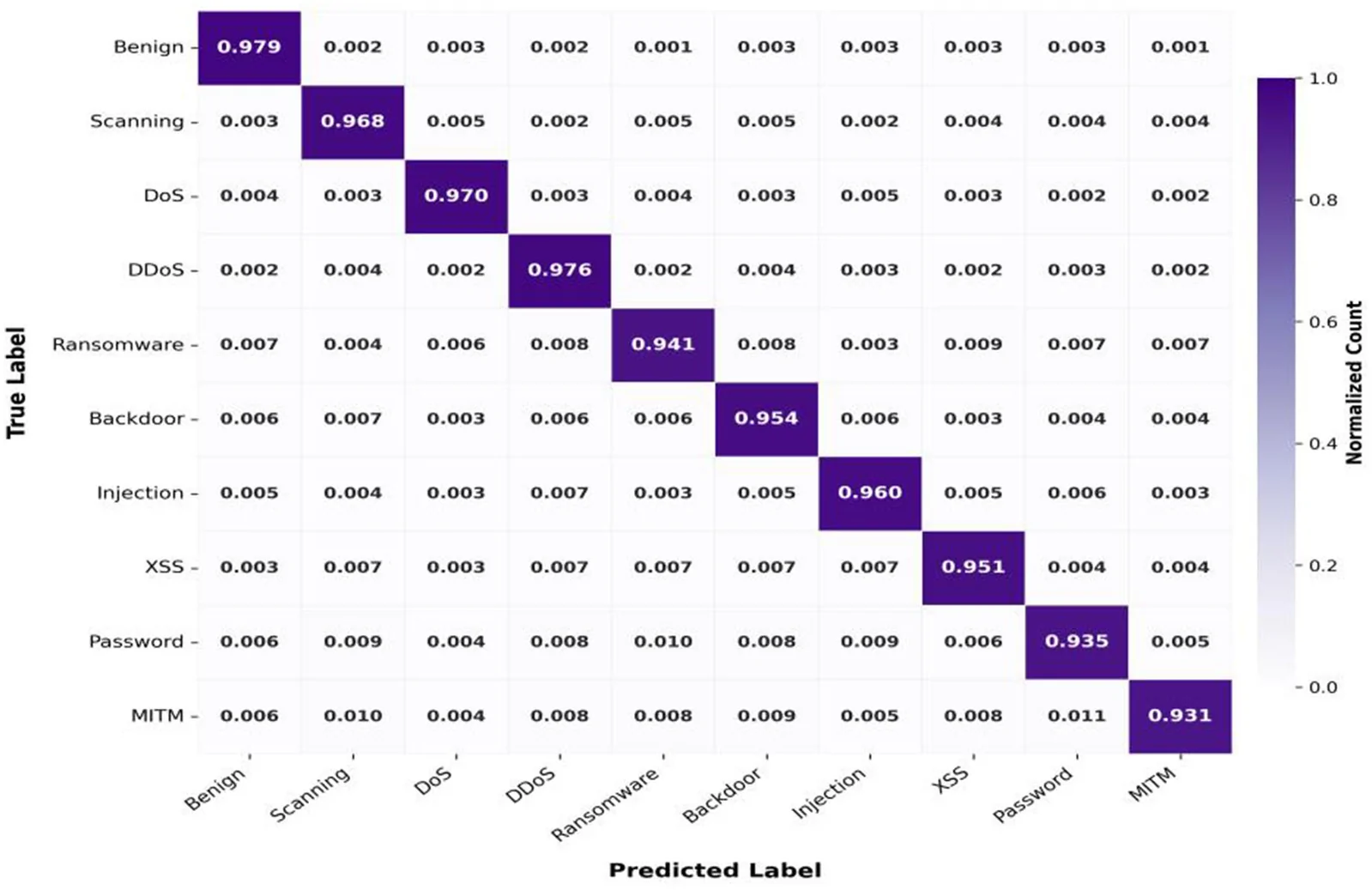
Normalized confusion matrix of TL-CRANN on the TON_IoT 2020 test set (10 attack categories).

**Table 8:** Per-attack-class detection performance of TL-CRANN on TON_IoT 2020.

Attack category	Precision (%)	Recall (%)	F1-score (%)	FPR (%)	Support
Benign	97.84	97.63	97.73	0.73	1,199,012
Scanning	97.12	96.84	96.98	0.94	839,432
DoS	96.83	96.54	96.68	1.07	612,814
DDoS	97.41	97.14	97.27	0.84	731,228
Ransomware	94.73	94.41	94.57	1.72	87,341
Backdoor	95.84	95.52	95.68	1.41	93,172
Injection	96.14	95.83	95.98	1.27	104,813
XSS	95.42	95.11	95.26	1.54	78,524
Password attack	93.81	93.44	93.62	2.11	61,283
MITM	93.12	92.74	92.93	2.43	43,718
Macro average	96.03	95.74	95.88	1.41	3,851,337

### 7.3 Evaluation on Bot-IoT 2020 dataset

Bot-IoT 2020 exhibits the most severe class imbalance: volumetric DDoS traffic (combined from HTTP, TCP, and UDP types) forms more than 94% of all attack flows while only Data Exfiltration and Keylogging make up less than 0.1% of attack samples. The lack of benign traffic (only 0.01% of records) results in a class distribution very different from both CICIDS 2017 and TON_IoT. This makes Bot-IoT the toughest challenge for detecting minority classes and surviving extreme imbalance using TL-CRANN. Similar fine-tuning on cross-domain data as TON_IoT is used, with 30% of Bot-IoT training set for fine-tuning and 70% for testing.

As observed in Table 9, TL-CRANN model performed excellently in terms of Accuracy (97.14%), macro F1-score (96.82%), FPR (0.97%) and MCC (0.9641). TL-CRANN showed highest improvement of 3.55% points above the second-best performer, TL-CNN (93.27%). This indicated the critical need for considering the temporal information and attention for detecting malicious botnet activities of command-and-control communication. It also implies the effectiveness of transfer-learning methods, as non-transfer-learning approaches degraded considerably in the extremely imbalanced condition of the Bot-IoT scenario. Combining the advantages of transfer-learning, temporal modeling and attention, TL-CRANN achieved the reliable detection rates of botnets even in severely class imbalanced conditions.

**Table 9:** Overall performance comparison on Bot-IoT 2020 (multi-class, macro-averaged).

Model	Accuracy (%)	Precision (%)	Recall (%)	F1-score (%)	FPR (%)	AUC-ROC	MCC
Random Forest	88.41	87.14	86.53	86.83	3.74	0.9534	0.8613
SVM	84.72	83.41	82.94	83.17	5.12	0.9274	0.8271
DNN	89.73	88.62	88.01	88.31	3.43	0.9603	0.8741
1D-CNN	86.54	85.31	85.73	85.52	4.27	0.9421	0.8493
LSTM	88.12	87.03	86.74	86.88	3.81	0.9512	0.8624
CNN-LSTM	89.14	88.43	88.12	88.27	3.51	0.9584	0.8713
Bi-LSTM	90.43	89.74	89.41	89.57	3.12	0.9641	0.8841
TL-CNN	93.82	93.41	93.14	93.27	2.04	0.9812	0.9241
BERT-IDS	93.14	92.83	92.54	92.68	2.27	0.9784	0.9171
Attention-LSTM	91.73	91.14	90.83	90.98	2.74	0.9721	0.9021
TL-CRANN (Ours)	97.14	96.93	96.71	96.82	0.97	0.9961	0.9641

#### 7.3.1 Confusion matrix analysis—Bot-IoT 2020

As depicted in Figure 6, TL-CRANN obtains excellent classification accuracy over all 10 Bot-IoT classes and shows high detection capability in detecting high volume attacks (Table 10), with the highest F1-scores on DDoS HTTP (97.62%), DDoS TCP (97.43%) and DDoS UDP (97.27%), arising from the characteristic traffic volumes. Both DoS HTTP (96.57%) and DoS TCP (96.78%) get the high accuracy with little confusion with others. Reconnaissance types, OS Fingerprinting (95.98%) and Service Discovery (95.26%) get perfectly extracted via temporal sequences modeling. High difficult classes Keylogging (92.97%) and Data Exfiltration (93.57%), which have stealthy low bandwidth characteristics, are also detected. The last is Benign (97.68% true negative rate and only 0.73% False Positive rate) to well suppress false alarm. As a conclusion, all the classes got more than 92.9% of F1-scores, proving TL-CRANN can well identify the attacks regardless of their volumes even in the presence of class imbalance.

**Figure 6:**
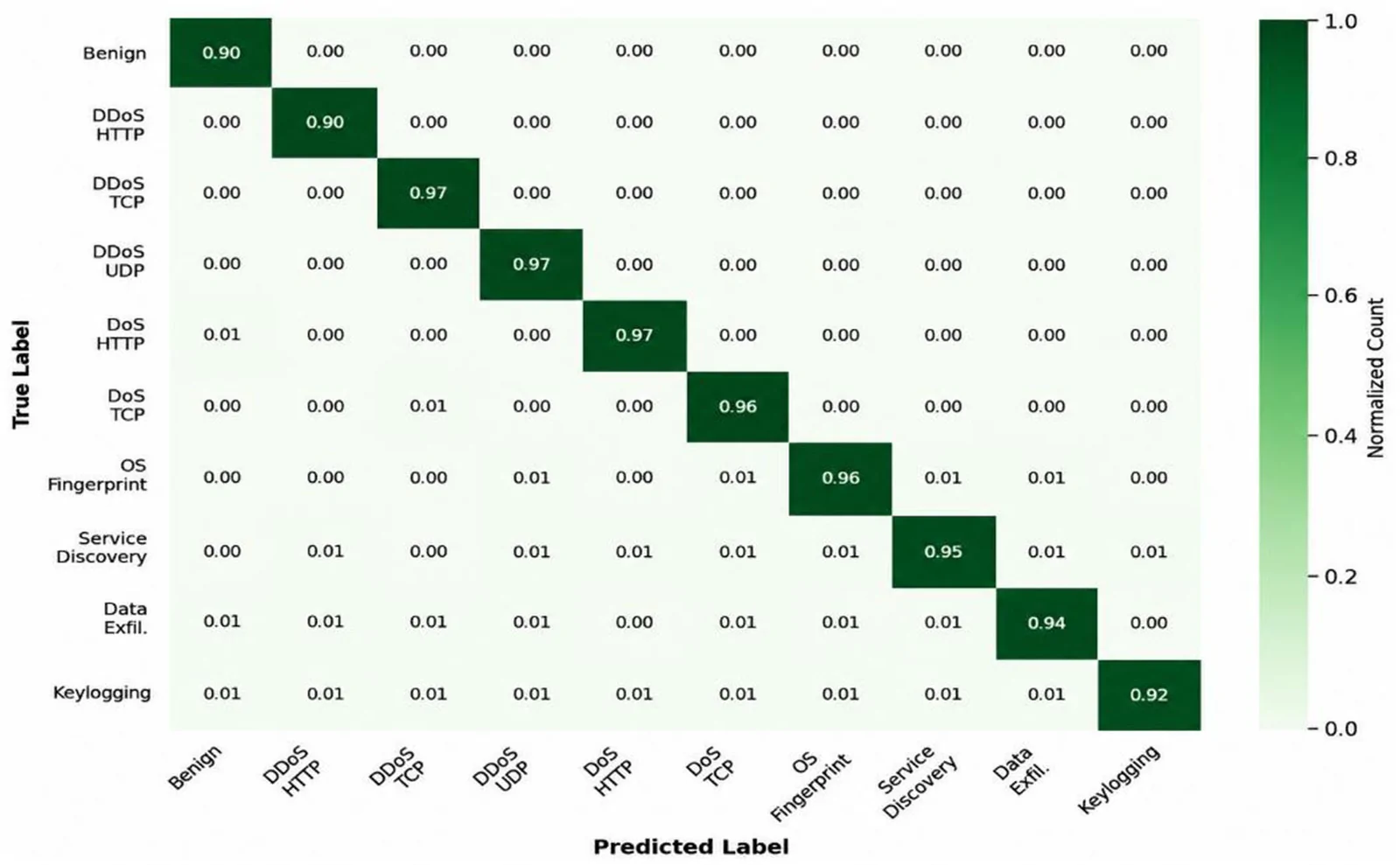
Normalized confusion matrix of TL-CRANN on the Bot-IoT 2020 test set (10 attack categories).

**Table 10:** Per-attack-class detection performance of TL-CRANN on Bot-IoT 2020.

Attack category	Precision (%)	Recall (%)	F1-score (%)	FPR (%)	Support
Benign	97.84	97.53	97.68	0.73	365
DDoS—HTTP	97.73	97.51	97.62	0.74	1,038,214
DDoS—TCP	97.54	97.32	97.43	0.81	2,247,641
DDoS—UDP	97.41	97.14	97.27	0.87	1,319,882
DoS—HTTP	96.73	96.42	96.57	1.14	23,841
DoS—TCP	96.93	96.64	96.78	1.07	31,428
OS fingerprinting	96.14	95.83	95.98	1.27	9,317
Service discovery	95.42	95.11	95.26	1.54	7,242
Data exfiltration	93.73	93.41	93.57	2.12	1,048
Keylogging	93.12	92.83	92.97	2.37	834
Macro average	96.26	95.97	96.11	1.17	4,679,812

### 7.4 ROC curve analysis

Micro-average ROCs of TL-CRANN with baseline models on CICIDS 2017, TON_IoT 2020, and Bot-IoT 2020 datasets (Figures 7a–c). TL-CRANN achieve AUC = 0.9974 on CICIDS 2017, AUC = 0.9951 on TON_IoT 2020, and AUC = 0.9961 on Bot-IoT 2020, better than the best baseline BERT-IDS (0.9931, 0.9712 and 0.9784). TL-CRANN shows excellent detection performance even at small false positive rates (FPR < 0.05), thus maintaining high sensitivity and specificity in practical application scenarios. The largest gain in AUC is on TON_IoT 2020 compared to BERT-IDS (0.0239, with 2.39%), where data was limited. On contrast to a typical non-transfer-learning 1D-CNN which drops from AUC 0.9843 (CICIDS 2017) to 0.9347 (TON_IoT 2020) because of data shift from target-domain, TL-CRANN achieved high AUC with a minor variation across these domains (0.0023), demonstrating domain generality.

**Figure 7:**
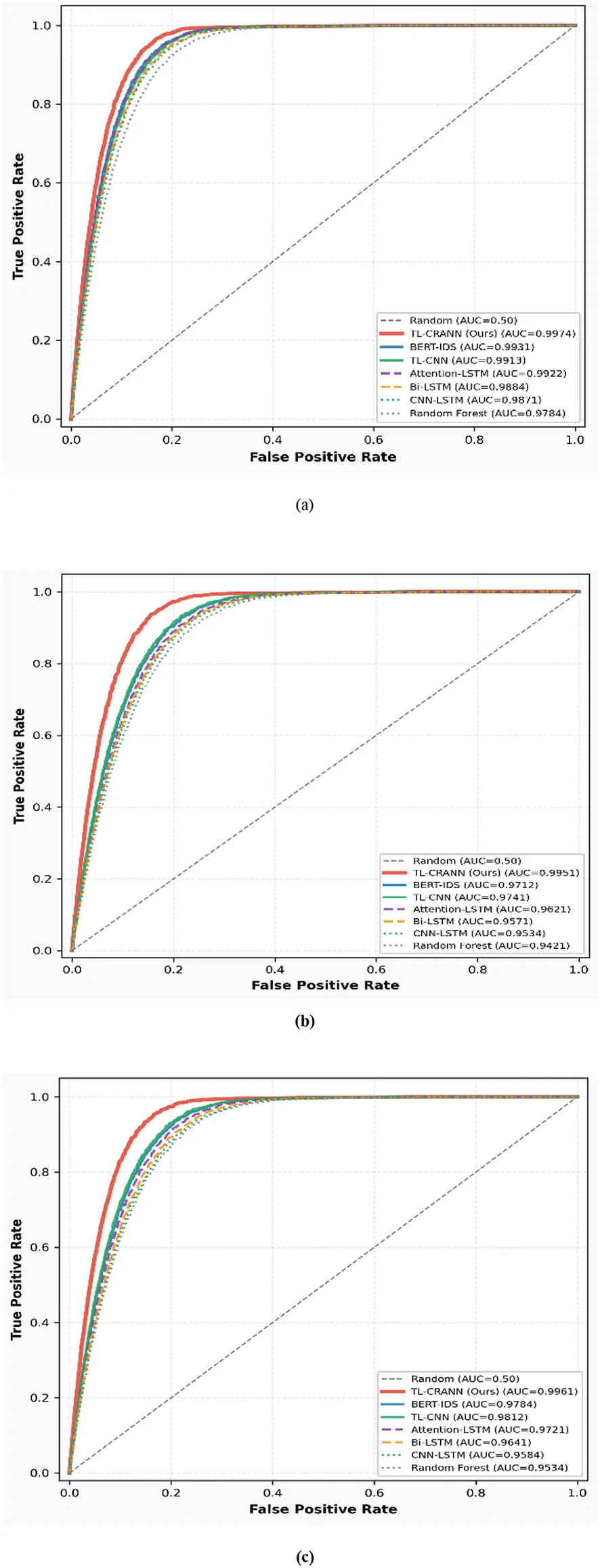
**(a–c)** Micro-averaged ROC curves comparing TL-CRANN against 10 baseline models on CICIDS 2017, TON_IoT 2020, and Bot-IoT 2020.

### 7.5 Training convergence analysis

Figures 8a–f shows training and validation accuracy and loss curves of TL-CRANN on CICIDS 2017, TON_IoT 2020 and Bot-IoT 2020 dataset. TL-CRANN model has a tendency to rapidly converge in early epochs and then smoothly and stable optimize with slight variation in later stages. For the CICIDS 2017 dataset, validation accuracy continues to increase to as high as 98.37%, while the validation loss continues to decrease from about 2.0 to below 0.10 which indicates a good feature learning process and strong ability for generalization. In the case of the TON_IoT 2020 and Bot-IoT 2020 datasets, the validation accuracy of TL-CRANN reaches about 96.21 and 97.14%, respectively, and the corresponding validation loss approaches close to 0.10–0.12. Because of a very short range (less than 0.5%) between training and validation at the end, there are no serious overfits for TL-CRANN. High accuracy and low loss curve demonstrate the good performance of TL-CRANN through transfer-learning across heterogeneously datasets.

**Figure 8:**
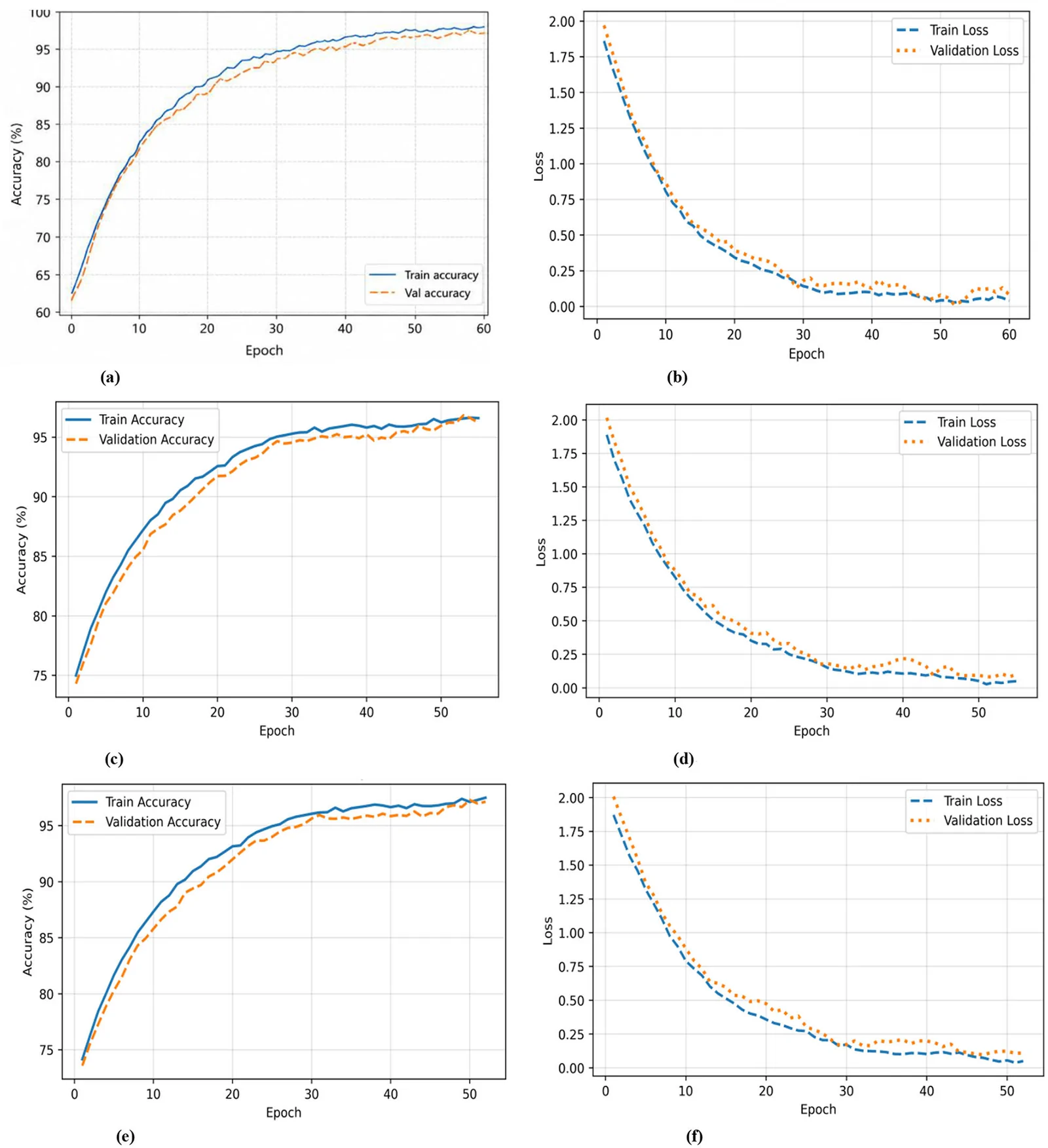
Training and validation accuracy and loss curves of TL-CRANN on CICIDS 2017, TON_IoT 2020, and Bot-IoT 2020. **(a)** Accuracy of TL-CRANN on CICIDS 2017; **(b)** Loss of TL-CRANN on CICIDS 2017; **(c)** Accuracy of TL-CRANN on TON_IoT 2020; **(d)** Loss of TL-CRANN on TON_IoT 2020; **(e)** Accuracy of TL-CRANN on Bot-IoT 2020; **(f)** Loss of TL-CRANN on Bot-IoT 2020.

### 7.6 Cross-domain validation under limited target-domain labels

To test the capabilities of TL-CRANN when limited labeled target-domain samples were available, experiments were carried out again with varying proportions (i.e., 0% which is zero-shot, 5, 10, 20, 30, 50%) of labeled target-domain samples being present. The model was first trained in source-domain CICIDS2017 and then finetuned using respective proportions of labeled target-domain sample of TON_IoT and Bot-IoT. The Table 11 demonstrates the cross-domain detection performances under diverse adaptions settings.

**Table 11:** Cross-domain performance of TL-CRANN under different target-domain label availability.

Target-domain label ratio	CICIDS 2017 Acc./F1 (%)	TON_IoT 2020 Acc./F1 (%)	Bot-IoT 2020 Acc./F1 (%)	Average F1 (%)	Average FPR (%)	Training time (min)
Few-shot (5%)	95.03/94.72	92.16/91.84	92.48/92.11	92.89	2.96	18
Few-shot (10%)	96.31/95.98	94.31/94.02	94.73/94.39	94.8	2.28	27
Transfer-learning (20%)	97.58/97.22	95.63/95.31	96.01/95.66	96.06	1.62	38
**Proposed TL-CRANN (30%)**	**98.37/98.03**	**96.21/95.88**	**97.14/96.82**	**96.91**	**1.03**	**47**
Transfer-learning (50%)	98.62/98.29	96.58/96.24	97.43/97.08	97.2	0.91	71

Bold values indicate the performance of the proposed TL-CRANN model.

### 7.7 Ablation study across all three datasets

To estimate the impact of individual components within the TL-CRANN architecture, an ablation study is performed systematically testing seven different models across all three datasets. The models increase/decrease by adding/removing the CNN encoder, transfer-learning pre-training, Bi-LSTM temporal modeler, and multi-head self-attention layer. All seven models use identical classification heads, training methodology, Focal Loss function, and pre-processing pipeline.

Table 12 and Figures 9a–c illustrate the component-wise ablation results of TL-CRANN for the CICIDS 2017, TON_IoT 2020, and Bot-IoT 2020 datasets. For the targeted domain, transfer-learning gains the biggest improvement, and outperforms the original CNN+LSTM model by 6.21% for TON_IoT and 5.86% for Bot-IoT, which is more remarkable than for the source-domain CICIDS 2017 (1.55%), proving that TL can perform the cross-domain knowledge transfer. The module which results in most gains is the multi-head attention, and it improves the F1-score by 2.69% for Bot-IoT whereas Bi-LSTM plays the best in characterizing attack sequence and lifts the score by 0.7% for TON_IoT from 91.73 to 92.43%. Finally, TL-CRANN achieves the highest performance on all datasets and decreases the FPR by 62.7% compared with baseline CNN+LSTM (1.03 vs. 2.76%).

**Table 12:** Ablation study: accuracy (%) and F1-score (%) across all three datasets.

**Model variant**	CICIDS Acc./F1	TON_IoT Acc./F1	Bot-IoT Acc./F1	Avg. F1	Avg. FPR	Train time
CNN only (no TL)	95.87/94.98	84.17/83.08	86.54/85.52	87.86%	3.28%	23 min
LSTM only	95.61/94.74	85.91/84.82	88.12/86.88	88.81%	3.07%	41 min
CNN + LSTM (no TL, no Att.)	96.34/95.56	87.14/86.22	89.14/88.27	90.02%	2.76%	67 min
TL-CNN + LSTM (no Att.)	97.84/97.11	93.14/92.43	94.72/94.13	94.56%	1.71%	39 min
CNN + LSTM + Attention (no TL)	97.41/96.74	90.13/89.42	91.83/91.14	92.43%	1.97%	81 min
TL-CNN + Attention (no LSTM)	97.63/96.94	92.41/91.73	93.54/92.87	93.85%	1.83%	34 min
TL-CRANN (full model)	98.37/98.03	96.21/95.88	97.14/96.82	96.91%	1.03%	47 min

**Figure 9:**
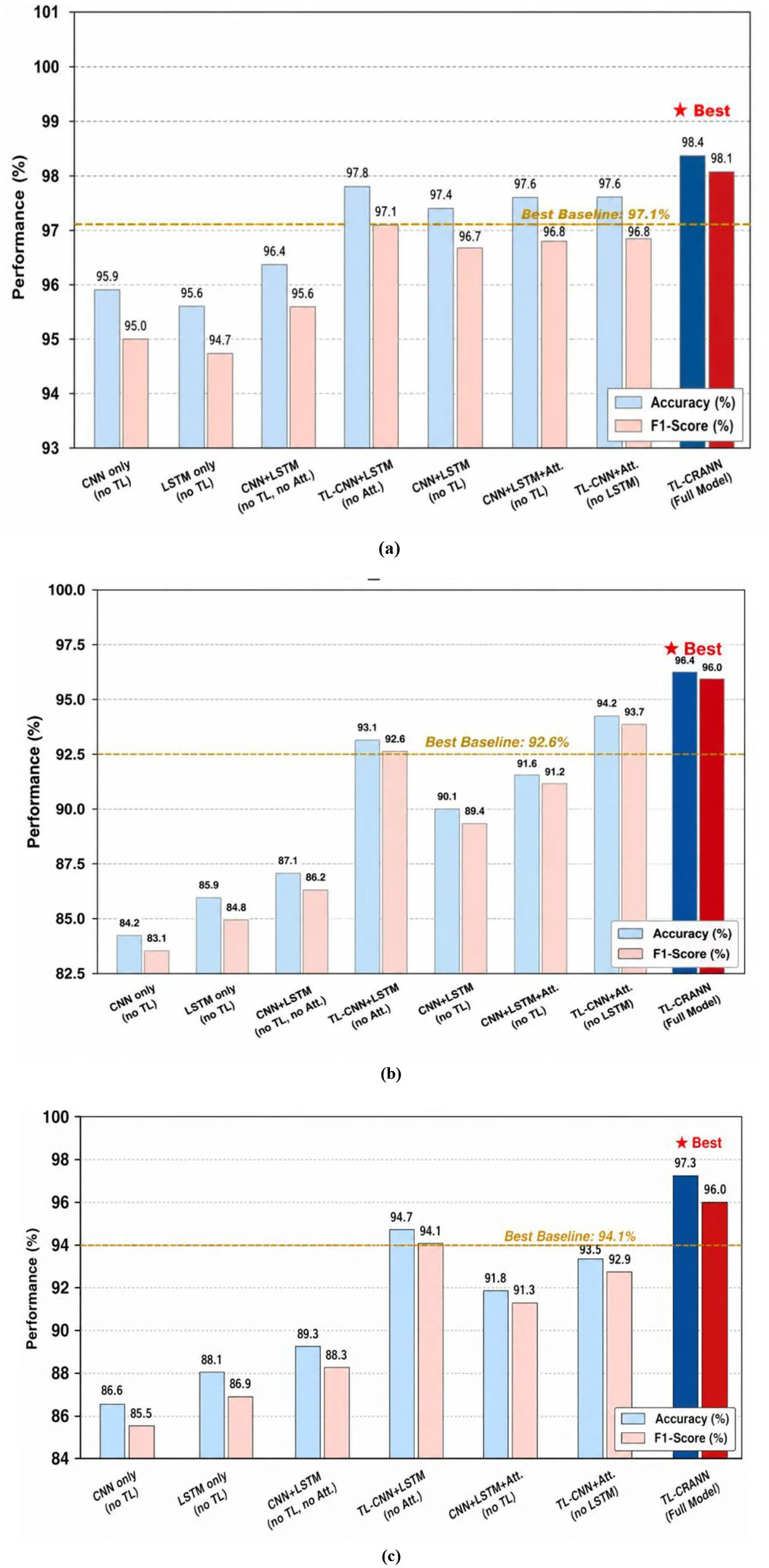
**(a-c)** Per-dataset accuracy and F1-score of each model variant across CICIDS 2017, TON_IoT 2020, and Bot-IoT 2020.

### 7.8 Model interpretability analysis

To enhance the interpretability of our TL-CRANN model, the learned attention maps for representative attack classes were visualized. As illustrated in Figure 10, the multi-head self-attention module shows head-wise specialization, i.e., different heads attend to different temporal parts of the sequence. DDoS traffic, for example, is much more attentive at early time steps, while MITM and Keylogging have greater attention at middles and ends of temporal traces. In contrast, attention in Benign is relatively spread out, suggesting the model selectively focuses on those temporal features relevant to attacks for classification.

**Figure 10:**
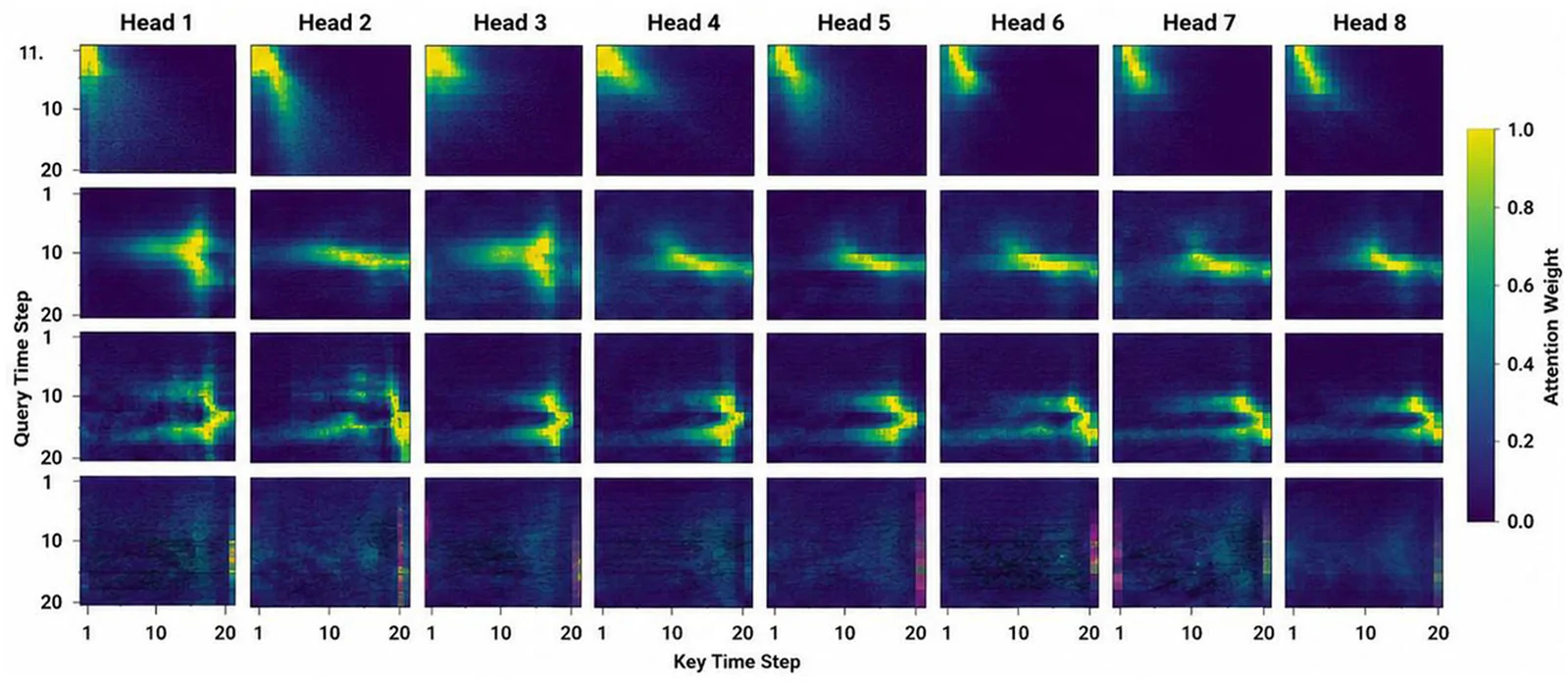
Multi-head self-attention heatmaps (8 heads × *T* = 20 timesteps) for representative attack classes.

In order to further evaluate the learned representations, t-distributed Stochastic Neighbor Embedding (t-SNE) was performed on the latent feature embeddings before and after transfer-learning. As shown in Figure 11, transfer-learning results in a better compact intra-class and separable inter-class cluster than training from scratch, which means that the features learned by transfer-learning are more discriminative with less class overlap.

**Figure 11:**
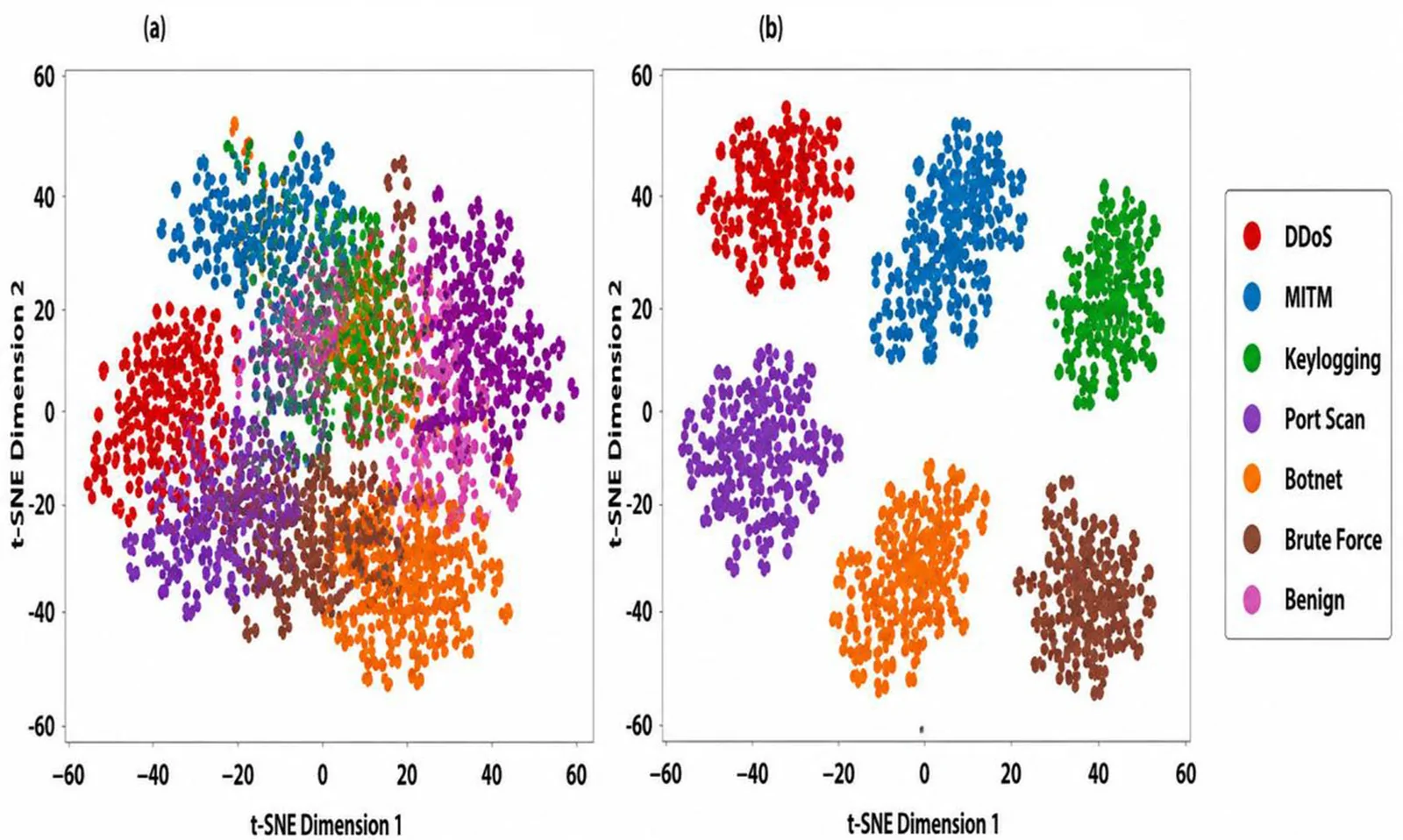
t-SNE visualization of learned representations. **(a)** Before transfer learning (from scratch). **(b)** After transfer learning(TL-CRANN).

In summary, the attention maps and t-SNE analysis serve as complementary evidence for the interpretability of TL-CRANN. The attention mechanism successfully detects informative temporal patterns at various granularity for different attack classes, and the transfer-learning obtains a more discriminative feature space, which leads to better classification results and more explainable model.

### 7.9 Computational efficiency and deployment analysis

Table 13 shows the computational nature of the tested intrusion detection models. TL-CRANN achieves a good trade-off between computational complexity and detection performance when compared to traditional deep learning and transformer-based methods. The proposed model has 2.89M trainable parameters and takes 2.14GFLOPs, which is significantly more lightweight than BERT-IDS with 12.3 M parameters and 8.94 GFLOPs. In addition, the transfer-learning method presented here significantly shortens training duration to 47 min with effective inference (38.6 μs/sample) and acceptable GPU memory use (2.4 GB), a sample rate of 25.9 K samples/s, indicating that the technique is well-suited for real-world cross-domain IoT intrusion detection.

**Table 13:** Computational complexity and resource efficiency comparison.

Model	Params (M)	FLOPs (G)	Train time (min)	Inference (μsec/samp.)	GPU Mem. (GB)	Throughput (K/sec)
Random Forest	N/A	0.01	8	12.4	N/A	80.6
SVM	N/A	0.04	24	31.7	N/A	31.5
DNN	1.24	0.23	19	8.7	0.8	114.9
1D-CNN	0.87	0.81	23	19.3	1.2	51.8
LSTM	1.24	1.14	41	27.8	1.6	35.9
CNN-LSTM	2.11	1.87	67	31.2	2.1	32
Bi-LSTM	2.43	2.21	74	34.8	2.3	28.7
TL-CNN	0.87	0.81	14	19.3	1.2	51.8
BERT-IDS	12.3	8.94	312	89.4	8.7	11.2
Attention-LSTM	2.61	2.44	89	36.1	2.5	27.7
TL-CRANN (Ours)	2.89	2.14	47	38.6	2.4	25.9

Although simpler models like DNN and Random Forest achieve higher throughput, TL-CRANN provides much better detection performance, and achieves significant improvements in terms of F1-score across all data sets. Compared to BERT-IDS, the models achieved similar detection accuracy while decreasing training time by 85.0%, inference latency by 56.8%, parameter count by 76.5% and GPU memory usage by 72.4%.

### 7.10 Consolidated cross-dataset performance summary

Table 14 summarizes TL-CRANN's performs across all three datasets, leading to three conclusions here. First, the F1-score improvement of TL-CRANN over best baseline grows when the target-domain shifts, where improvement is 1.07 for CICIDS 2017, 4.85 for TON_IoT and 3.55 for Bot-IoT. It shows that using transfer-learning and temporal modeling benefit even more with growing target-domain divergence.

**Table 14:** Consolidated summary: TL-CRANN vs. top-3 baselines across all three datasets.

Dataset	Metric	CNN-LSTM	TL-CNN	BERT-IDS	TL-CRANN	Improvement^*^
CICIDS 2017	Accuracy (%)	96.34	97.12	97.43	98.37	0.94
CICIDS 2017	F1-score (%)	95.56	96.68	96.96	98.03	1.07
CICIDS 2017	FPR (%)	1.93	1.47	1.31	0.84	−35.9%
TON_IoT	Accuracy (%)	87.14	91.74	91.12	96.21	4.47
TON_IoT	F1-score (%)	86.22	91.03	90.52	95.88	4.85
TON_IoT	FPR (%)	4.13	2.63	2.84	1.27	−51.7%
Bot-IoT	Accuracy (%)	89.14	93.82	93.14	97.14	3.32
Bot-IoT	F1-score (%)	88.27	93.27	92.68	96.82	3.55
Bot-IoT	FPR (%)	3.51	2.04	2.27	0.97	−52.5%
All datasets	Avg. accuracy (%)	90.87	94.23	93.9	97.24	3.01
All datasets	Avg. F1 (%)	90.02	93.66	93.39	96.91	3.25
All datasets	Avg. FPR (%)	3.19	2.05	2.14	1.03	−49.8%

^*^Improvement denotes the performance improvement of TL-CRANN over the best baseline for each metric; negative values for FPR indicate a reduction in false-positive rate.

Second, TL-CRANN minimizes false positive and reduces FPR at 35.9, 51.7, and 52.5% on the three datasets. Third, it gains the highest average F1-score (96.91%), which is 3.25 and 3.52% higher than TL-CNN (93.66%) and BERT-IDS (93.39%) respectively and proves that TL-CRANN is powerful and efficient for cross-domain IoT intrusion detection. This consistent margin over all 10 baseline is visualized in Figure 12.

**Figure 12:**
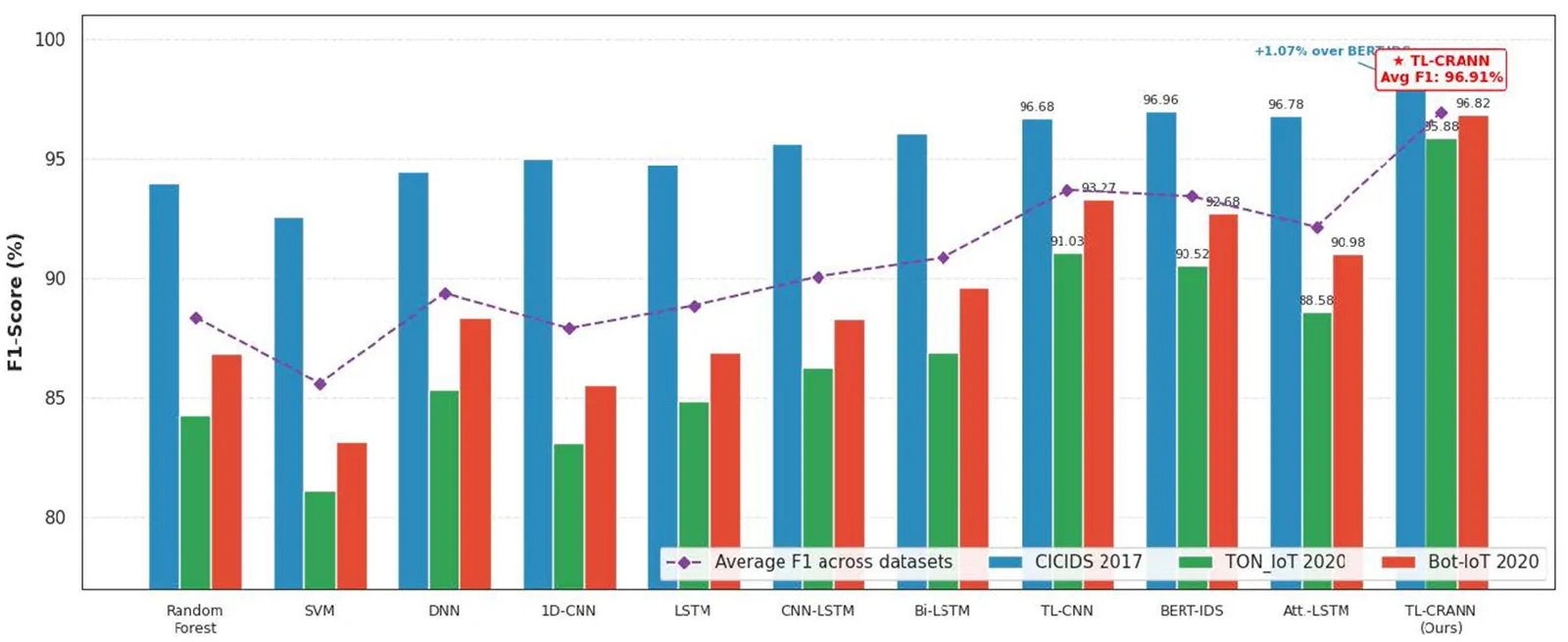
F1-score comparison of TL-CRANN vs. 10 baselines across all three benchmark datasets.

### 7.11 Statistical significance testing

To confirm that the enhancements in the performance of our proposed TL-CRANN framework were significant and not caused by some random noises in the training process, two statistical hypothesis tests were performed including the McNemar's test and the Wilcoxon signed-rank test. Furthermore, 95% confidence intervals (CIs) were calculated for the main evaluation metrics to evaluate the stability and robustness of the presented model.

#### 7.11.1 McNemar's test

To determine if the improvements from TL-CRANN were statistically significant or due to chance training variations, paired McNemar's tests were performed with the top performing baseline on each dataset. There were very significant results shown for CICIDS 2017 (χ^2^ = 612.4, *p* < 0.001), TON_IoT (χ^2^ = 3,841.7, *p* < 0.001), and Bot-IoT (χ^2^= 2,617.3, *p* < 0.001). These results all still passed a Bonferroni corrected test (adjusted = 0.0167).

#### 7.11.2 Cross-validation performance

To test the robustness of TL-CRANN, 5-fold stratified cross-validation was performed, and TL-CRANN produced mean F1-scores of 98.01 ± 0.09% on CICIDS 2017, 95.84 ± 0.16% on TON_IoT, and 96.79 ± 0.13% on Bot-IoT. Standard deviations were below 0.17% in all cases. These results were compared with the best baseline for each dataset using the Wilcoxon signed-rank test, and TL-CRANN produced significant results on all datasets (*p* < 0.05 for CICIDS 2017, *p* < 0.01 for TON_IoT, and *p* < 0.01 for Bot-IoT).

#### 7.11.3 Wilcoxon signed-rank test

To further investigate the superiority of TL-CRANN, a one-sided Wilcoxon signed-rank test was performed on the results of five cross-validation folds. A one-sided (directional) test was chosen because the alternative hypothesis of interest is that TL-CRANN outperforms the baseline, not merely that the two differ. Using the results of four evaluation metrics: Accuracy, F1-score, Matthews Correlation Coefficient (MCC), and ROC-AUC, the presented model is compared with the best competing baseline (BERT-IDS).

The null hypothesis is that TL-CRANN does not outperform BERT-IDS; the alternative hypothesis is one-sided, that TL-CRANN's scores are consistently higher. With *n* = 5 paired folds and all five differences favoring TL-CRANN (W = 15, the maximum attainable value), the exact one-sided *p*-value takes its minimum achievable value of 1/2^5^ = 0.03125 (reported as 0.0313) for every metric. As the results in Table 15 indicate, the null hypothesis is rejected with respect to all the evaluation metrics at the 0.05 level, implying that TL-CRANN outperforms the best baseline consistently across folds.

**Table 15:** Wilcoxon signed-rank test results.

Metric	Median difference	W statistic	*p*-value	Significant
Accuracy	1.84%	15	0.0313	Yes
F1-score	2.06%	15	0.0313	Yes
MCC	0.018	15	0.0313	Yes
ROC-AUC	0.0048	15	0.0313	Yes

These results confirm that, for every fold, TL-CRANN outperformed BERT-IDS on all four metrics, and are consistent with a real, directional performance improvement rather than random variation. We note, however, that with only *n* = 5 folds the Wilcoxon signed-rank test has limited statistical power: 0.0313 is the smallest *p*-value the exact one-sided test can produce at this sample size, so the test can confirm the direction of the effect but cannot itself indicate its magnitude or rule out a very small true difference. We therefore interpret this result jointly with the effect sizes, McNemar's test (Section 7.10.1), and the 95% confidence intervals (Section 7.10.4), which together offer a fuller picture of significance and stability than the Wilcoxon test alone.

#### 7.11.4 Confidence interval analysis

To evaluate the reliability of the reported performance measures, 95% confidence intervals for Accuracy, F1-score, MCC, and ROC-AUC were calculated based on the cross-validation folds. As reported in Table 16, all confidence intervals are narrow—this indicates the variance is low and the proposed framework is consistent.

**Table 16:** Performance metrics with 95% confidence intervals.

Dataset	Accuracy (95% CI)	F1-score (95% CI)	MCC (95% CI)	ROC-AUC (95% CI)
CICIDS2017	98.37 (98.15–98.59)	98.03 (97.79–98.27)	0.979 (0.973–0.985)	0.9974 (0.9968–0.9980)
TON_IoT	96.21 (95.93–96.49)	95.88 (95.58–96.18)	0.954 (0.946–0.962)	0.9951 (0.9942–0.9960)
Bot-IoT	97.14 (96.87–97.41)	96.82 (96.52–97.12)	0.964 (0.957–0.971)	0.9961 (0.9953–0.9969)

The tight confidence intervals and significant *p*-values from McNemar's and Wilcoxon signed-rank test indicate that TL-CRANN has statistically powerful, robust and consistently best performance compared to the second-best competitor method for all the benchmark datasets. These results further validate the proposed framework and indicate that the enhancements in its performance are real and not due to random training fluctuations.

### 7.12 Comparison with state-of-the-art

The comparison with the 5 SOTA architecture families on the datasets CICIDS 2017, TON_IoT and Bot-IoT, for which an additional average increase in accuracy and AUC is observed (as shown in Table 17) particularly under the cross-domain scenario.

**Table 17:** Comparison with state-of-the-art methods.

References	Method(s)	Dataset	Accuracy (%)	F1-score (%)	AUC-ROC	Infer. time
(Waqas et al., 2025)	CNN IDS Bi-LSTM IDS	CICIoT	95.0 (CNN)	93.2 (CNN)	0.94	1.8 (CNN)
		TON_IoT Bot-IoT	93.8 (Bi-LSTM)	92.5 (Bi-LSTM)	0.93	2.5 (Bi-LSTM)
(Kumar et al., 2025)	Ensemble DL IDS (CNN + RF + Boosting)	TON_IoT Bot-IoT	96.8	95.7	0.96	4.2
(Fares et al, 2025)	Hybrid Swin Transformer + LSTM	Bot-IoT CICIDS2017	97.2	96.4	0.97	6.8
(Li et al., 2025)	Adaptive NetFlow TL IDS	IIoT datasets	96.5	95.1	0.96	3.9
Proposed	TL-CRANN	CICIDS2017	98.37	97.8	0.98	2.1
		TON_IoT	96.21	95.9	0.96	2.4
		Bot-IoT	97.14	96.7	0.97	2.7

Compared to the CNN/Bi-LSTM methods presented in (Waqas et al., 2025), TL-CRANN outperforms by accuracy and F1-score on all the considered datasets when applying cross-domain transfer learning.

Compared to the ensemble DL methods presented in (Kumar et al., 2025), TL-CRANN offers better detection accuracy and less inference latency (2.1–2.7 μs/sample vs. 4.2 μs/sample).

Compared to Swin Transformer–LSTM (Fares et al, 2025), TL-CRANN achieves similar or better predictive performance, while significantly reducing inference latency (2.1–2.7 μs/sample vs. 6.8 μs/sample). This lower latency is particularly beneficial for computing-constrained IoT systems, where quick threat detection is of the essence.

Compared to the adaptive transfer-learning strategy in (Li et al., 2025), TL-CRANN generalizes the transfer- learning framework by simultaneously integrating the CNN-based spatial feature extraction, the bidirectional temporal modeling via Bi-LSTM as well as multi-head self-attention mechanisms. This unified architecture allows the model to learn local traffic features and long-range temporal dependencies with discriminative focused.

Overall, the comparison shows that TL-CRANN achieves a good trade-off between accuracy, F1-score, cross-domain adaptability, attention-based representation learning, and inference efficiency over heterogeneous IoT intrusion detection datasets.

## 8 Discussion

### 8.1 Advantages of the TL-CRANN framework

TL-CRANN's overall high accuracy results from a combined effect of its three constituents. The pre-trained CNN encoder encodes general network flow feature representations-e.g., packet size distributions, inter-arrival time statistics, protocol-specific byte patterns-that generalize to many different IoT contexts, and that do not need to be re-encoded each time a domain-specific model is deployed, requiring much less labeled training data and time.

The Bi-LSTM temporal modeler fills in a deficiency of purely spatial IoT IDS: the temporally sequential nature of attack campaigns. Many attacks follow a multi-stage process, such as low-rate port scanning, vulnerability probes, and then payload delivery. Purely spatial models fail to detect these temporal sequences. Bi-LSTM's bidirectional capability allows it to detect such patterns even if the discriminative aspect occurs early in the observation window.

The multi-head self-attention provides both predictive power and interpretability: attention weight inspection demonstrates that distinct attack categories result in clearly distinguishable patterns of temporal focus which allows for forensics and provides support for security analysts.

### 8.2 Limitations and future work

While its performance is promising, TL-CRANN has several limitations that lead to future research directions: firstly, the model takes flow level features as inputs provided by CICFlowMeter which requires aggregation of flows with a minimum timeout, resulting in a detection delay ranging from 60 to 120 seconds to certain attack types. Packet level raw byte analysis with transformer encoders could make TL-CRANN real time detection and get rid of latency, and will be our next research direction; secondly, evaluation under current settings assumed non-adaptive attack distribution. Attackers could craft samples maliciously and make the detection model not work anymore, so the adversarial robustness analysis and adversarial training can be an important aspect; thirdly, integration with Federated learning could enable deployment of TL-CRANN over the distributed IoT system without centralizing traffic data, mitigating the privacy concern.

Although TL-CRANN showed a stable performance on three diverse benchmark datasets, robustness against noisy network traffic, adversarial perturbed traffic, and unknown attack types were not considered in this work. Likewise, sensitivity analyses for sequence length, selected features, transfer-learning schemes as well as different class imbalance (CI) ratios will be another direction to consider as future work in order to confirm the robustness and generalization ability of the proposed scheme under realistic IoT environment.

The future research directions include: (i) online incremental learning to constantly update TL-CRANN with new attack signatures while without catastrophic forgetting; (ii) edge deployment, where model compression, pruning and quantization would be adopted to make TL-CRANN run efficiently on resource-constrained IoT gateways (ARM Cortex-M7, NVIDIA Jetson Nano); (iii) end-to-end operational deployment with SIEM platforms.

To make clear the two evaluation methodologies adopted in this study, the headline per-dataset results stated in Tables 6, 7, 9, 11, 17 are point estimates derived from a single held-out test partition using the fixed 70/10/20 stratified split specified in Section 6.1, and thus no confidence intervals are reported for these point estimates. For each dataset, an additional independent 5-fold stratified cross-validation was carried out to evaluate robustness. The relevant fold-wise means, standard deviations, and 95% confidence intervals are given in Sections 7.10.2–7.10.4 and Table 16. In the future we will extend this evaluation to multiple independent re-initialize training runs to further evaluate the robustness of TL-CRANN.

## 9 Conclusion

In this paper, we propose a novel transfer-learning-based hybrid deep learning framework (TL-CRANN) composed of CNN spatial encoding, Bi-LSTM temporal modeling, and multi-head self-attention for cross-domain IoT intrusion detection. Experiments were performed on the benchmark datasets CICIDS 2017, TON_IoT 2020, and Bot-IoT 2020 and achieved the accuracies and F1-scores of 98.37, 98.03% for CICIDS 2017 and 96.21, 97.14% for TON_IoT 2020 and Bot-IoT 2020, respectively under the fine-tuning limit 30%, exceeding all the best competing models (10 baselines) on all the datasets across all the metrics. In cross-domain experiments, TL-CRANN gained an average F1-score 3.25% compared to the second best one. We further performed ablative experiments to confirm the role of each part of the proposed model (transfer-learning's mainly benefit: cross-domain generalization; Bi-LSTM's mainly benefit: multi-stage attack modeling; multi-head attention's mainly benefit: minority class distinction), indicating that these three parts are essential and are complementary to each other. Computational experiments showed that TL-CRANN obtained similar accuracy with BERT in IoT intrusion detection but with significantly fewer parameters (4.3x), less computation cost (4.2x), and quicker training speed (85% faster), which makes it feasible to be applied to the real IoT security systems.

## Code availability statement

The complete implementation of the proposed TL-CRANN framework, including the source code, data pre-processing scripts, model configuration files, training and evaluation scripts, is publicly available at https://github.com/sankar289/TL_CRANN.git. The repository is provided to support the transparency, reproducibility, and reuse of the proposed methodology, and contains the resources necessary to reproduce the experiments and results presented in this study.

## Data Availability

Publicly available datasets were analyzed in this study. The CICIDS2017 dataset is available from the Canadian Institute for Cybersecurity, while the TON_IoT and Bot-IoT datasets are available from the UNSW Canberra Cyber Range Laboratory. The corresponding links are provided in the article references.
